# Specular Electron Focusing between Gate-Defined Quantum Point Contacts in Bilayer Graphene

**DOI:** 10.1021/acs.nanolett.3c00499

**Published:** 2023-06-08

**Authors:** Josep Ingla-Aynés, Antonio L. R. Manesco, Talieh S. Ghiasi, Serhii Volosheniuk, Kenji Watanabe, Takashi Taniguchi, Herre S. J. van der Zant

**Affiliations:** † Kavli Institute of Nanoscience, Delft University of Technology, Lorentzweg 1, 2628 CJ Delft, The Netherlands; ‡ Research Center for Functional Materials, National Institute for Materials Science, 1-1 Namiki, Tsukuba 305-0044, Japan; § International Center for Materials Nanoarchitectonics, National Institute for Materials Science, 1-1 Namiki, Tsukuba 305-0044, Japan

**Keywords:** ballistic transport, bilayer graphene, quantum point contact, trigonal warping

## Abstract

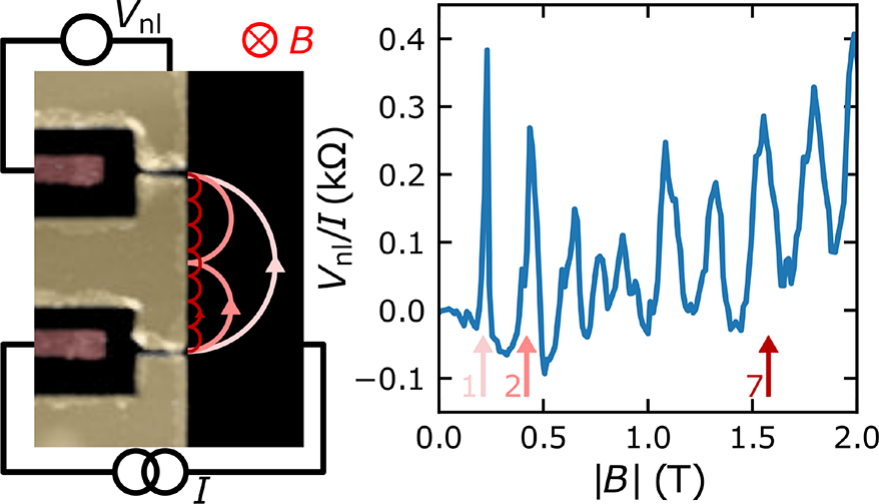

We report multiterminal measurements in a ballistic bilayer graphene (BLG) channel, where multiple spin- and valley-degenerate quantum point contacts (QPCs) are defined by electrostatic gating. By patterning QPCs of different shapes along different crystallographic directions, we study the effect of size quantization and trigonal warping on transverse electron focusing (TEF). Our TEF spectra show eight clear peaks with comparable amplitudes and weak signatures of quantum interference at the lowest temperature, indicating that reflections at the gate-defined edges are specular, and transport is phase coherent. The temperature dependence of the focusing signal shows that, despite the small gate-induced bandgaps in our sample (≲45 meV), several peaks are visible up to 100 K. The achievement of specular reflection, which is expected to preserve the pseudospin information of the electron jets, is promising for the realization of ballistic interconnects for new valleytronic devices.

Electronic devices with well-defined ballistic electron trajectories have triggered extensive research,^1−4^ and to exploit their full potential, specular reflection of electron jets is a major requirement. Electrostatically defined geometries are optimal platforms to realize the specular reflection, as shown by transverse electron focusing (TEF) measurements.^5−16^ In this context, the exceptional electronic properties of graphene make it an ideal candidate for a wide variety of gate-defined devices where Klein tunneling enables new functionalities.^2,17−20^ However, the absence of a bandgap complicates the creation of collimated beams and specular mirrors in graphene. The former has been realized by etching high-mobility graphene devices in absorptive pinhole collimators.^21^ The latter has been improved by recent fabrication progress, leading to the observation of multiple focusing peaks.^10,13^ However, the reflection induced by disordered graphene edges is not specular.^22^ This is a fundamental limitation that in TEF experiments results in a decrease of the peak amplitude as the number of reflections at the edge increases^9,10,12,13^ and randomizes the valley degree of freedom.^22^ An alternative approach has been implemented in the quantum Hall regime, where the gaps between Landau levels have been used to create gate-defined interferometers^23−25^ and quantum point contacts (QPCs).^25^ However, the effective confinement of carriers at zero magnetic field in monolayer graphene remains a challenge.

In contrast, bilayer graphene (BLG) is a tunable-bandgap semiconductor with a trigonally distorted Fermi surface.^26−30^ It has recently been introduced as an ideal system for the realization of gate-defined QPCs^31−39^ capable of transmitting valley-polarized electron jets^40^ and of hosting quantum dots with controllable spin and valley polarizations.^41,42^ Even though BLG hosts extraordinary properties, such as chirality-assisted cloaking^43,44^ or anti-Klein tunneling,^45^ experiments on gate-defined BLG devices have so far focused on the characterization of QPCs,^31−33,36−38,40^ quantum dots,^41,42,46^ quantum interference effects,^47^ and topological edge channels.^48−52^

In this work, we exploit the electrically tunable bandgap of BLG to create ballistic multiterminal BLG devices and measure TEF between gate-defined QPCs. We observe up to eight focusing peaks with comparable amplitudes, which is a clear indication of specular reflection at the gate-defined edges. Temperature-dependent measurements show that the TEF signal persists at up to 100 K.

We fabricated two double-gated, boron nitride (hBN)-encapsulated BLG heterostructures on few-layer graphene back gates, each containing multiple devices using the dry transfer technique described in refs (53 and 54). The electrodes were defined by using conventional e-beam lithography. The BLG flakes were connected to Ti/Au electrodes (brown rectangles in Figure 1a) after using a CHF_3_/O_2_ plasma to etch the upper hBN and BLG layers at the contact area.^55^ The top gates, which are dark yellow in Figure 1a, were deposited on the top hBN (see the Supporting Information (SI) Section S1 for the fabrication details). The side and top view images of a typical QPC are shown in Figure 1a. Here we discuss the results on the first heterostructure (sample 1); the results on sample 2 are shown in SI Section S9.

**Figure 1:**
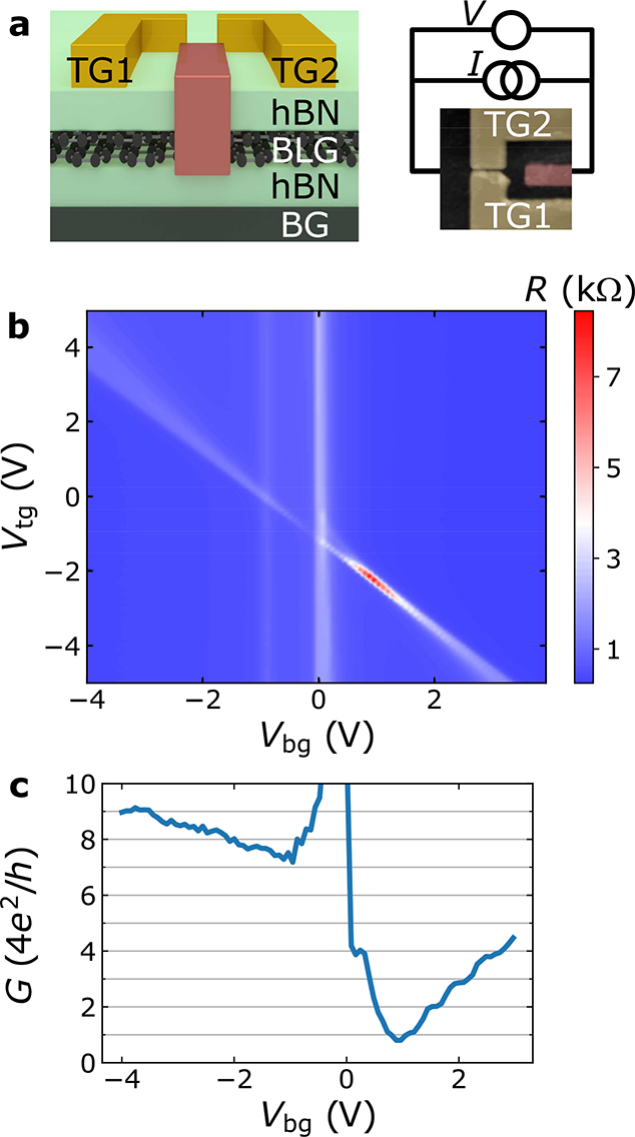
Gate-defined QPCs in the BLG at 1.8 K. (a) Side (left) and top (right) view of the fabricated device. The top view is a false-color AFM image. The separation between the split top gates (TG1 and TG2) is approximately 50 nm, and their width is 580 nm. At the side view (left panel), the hBN layers are green, and the BLG and the few-layer graphene back gate (BG) are black. In both panels, the contacts to the BLG flake are brown, and the top gates (TG) are dark yellow. (b) Two-terminal resistance (*R*) of one of the contacts used for the transverse electron focusing experiments as a function of *V*_bg_ and *V*_tg_. *V*_tg_ is the same for TG1 and TG2. (c) Point contact conductance obtained along the diagonal line in panel b that follows *V*_tg_ ≈ *V*_tg_^0^ – β*V*_bg_, corresponding to the charge neutrality point of the double-gated regions. *V*_tg_^0^ ≈ −1.08 V is the charge neutrality point at *V*_bg_ = 0, and β is the ratio between the back and top gate capacitances. The TEF measurements are also performed along this line.

The two-terminal resistance of the QPC, defined as *R* = *V*/*I*, where *V* and *I* are the measured voltage and applied current, respectively (Figure 1a, right panel), has been recorded as a function of the top gate voltage (*V*_tg_) and the back gate voltage (*V*_bg_). As shown in Figure 1b, three features can be distinguished from this result: The first one is a vertical line at *V*_bg_ ≈ 0, which corresponds to the charge neutrality point (CNP) of the non-top-gated BLG channel. The CNP does not occur at exactly *V*_tg_ = 0 due to small hole doping. The second feature is a faint vertical line at *V*_bg_ ≈ −1 V. Four-terminal measurements (see SI Section S3) indicate that it corresponds to the CNP of the BLG near the Ti/Au contacts, where the top hBN and BLG have been etched.

The last feature is a diagonal line that has a negative slope (*V*_tg_ decreases as *V*_bg_ increases) that corresponds to the CNP of the regions under TG1 and TG2. Because both *V*_bg_ and *V*_tg_ influence the carrier density (*n*) at these regions, the introduction of electrons by *V*_bg_ to the BLG channel must be counteracted by an opposite *V*_tg_ to keep the channel charge neutral. We use the slope of this line to obtain the ratio between the top gate (*C*_tg_) and back gate (*C*_bg_) capacitances: β = *C*_bg_/*C*_tg_ = −Δ*V*_tg_/Δ*V*_bg_ ≈ 1.22. This value is consistent with the factor of 1.22 obtained from the ratio between the hBN-flake thicknesses extracted from AFM imaging (see SI Section S1). Even though the electric field applied by the gates opens a bandgap in the double-gated BLG regions which increases with |*V*_bg_|,^26−29^ the resistance along the diagonal line does not increase with |*V*_bg_|. This is due to the small gap between TG1 and TG2 (Figure 1a). In this region the carrier density is not zero, leading to the formation of a *V*_bg_-controlled QPC with tunable carrier density.

To determine if the QPC conductance (*G*) is quantized, we have determined its resistance by taking, for each *V*_bg_, the difference between the maximal and the minimal *R*. This operation allows us to subtract the resistances of the Ti/Au contacts and the BLG regions that are not affected by *V*_tg_. The results are shown in Figure 1c. For negative *V*_bg_, *G* shows values higher than 7 × 4*e*^2^/*h* and changes in a monotonic way with small oscillations. In contrast, for positive *V*_bg_, *G* shows four steps at *G* = *N* × 4*e*^2^/*h* with *N* = 1, 2, 3, and 4. This behavior, which is reproduced in five of the six QPCs characterized, indicates the formation of a spin and valley-degenerate QPC.^33,36,37,56^ Note that the sharp increase of *G* near *V*_bg_ = 0 is a consequence of the extraction method when there is no bandgap under the double-gated regions, and *R* shows very small changes with *V*_tg_. Even though the reason for the electron–hole asymmetry is not clear, we believe that one possibility may be a residual doping of the double-gated regions caused by the fabrication. Because the QPC region is not affected by this process, the potential landscape could become asymmetric to the sign reversal of the gate voltages, modifying the confinement potential. This could make the QPC narrower for electron than hole doping or modify its carrier density. In addition, the electric field applied on the BLG at the CNP changes sign with *V*_bg_, leading to opposite layer polarizations^57^ which could also enhance the asymmetry.

When a magnetic field (*B*) is applied perpendicular to the plane of a ballistic BLG device, electrons deviate from their straight trajectories by the Lorentz force. If the Fermi surface is circular, they follow circular orbits with radius *r*_c_ = *ℏk*_F_/*eB*, where *ℏ* is the reduced Planck constant and *k*_F_ is the Fermi wavevector . As a consequence, the transmission between different contacts connected at a distance *L* from each other shows maxima at magnetic fields (*B*_f_) given by^5,7^
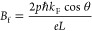
1where θ is the angle at which the electron flow departs from the emitter, *L* = 2 μm is the injector–detector distance, and *p* = 1, 2, 3, ..., *n* is an integer which accounts for the *p* – 1 reflections that occur at the device edge between the contacts (Figure 2a).

**Figure 2:**
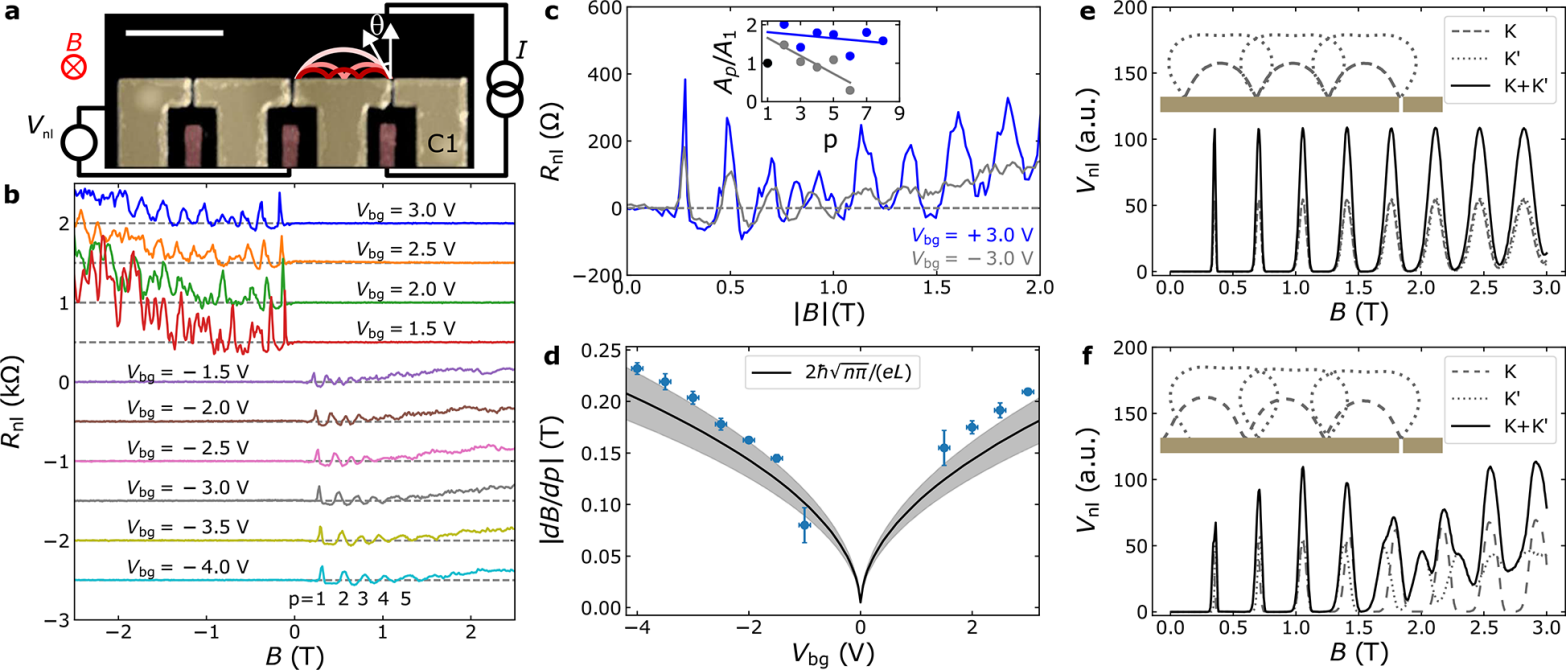
Transverse electron focusing between gate-defined QPCs in the BLG at 1.8 K. (a) Measurement geometry. The nonlocal voltage (*V*_nl_) is measured as a function of *B* while applying current *I* between the right QPC and a reference lead. The ballistic trajectories are sketched for the three first focusing peaks, which involve 0, 1, and 2 reflections with the gate-defined edge and assuming no trigonal warping. The scale bar is 2 μm. (b) Nonlocal resistance (*R*_nl_ = *V*_nl_/*I*) as a function of *B* for different *V*_bg_ values. The dashed lines show the spectra offsets, which have been introduced for clarity. *V*_tg_ is tuned to follow the charge neutrality line of the top-gated regions (diagonal line in Figure 1b). (c) Focusing spectra extracted from panel b at *V*_bg_ = ±3 V. A small offset in *B* was added to correct for the magnet remanence. The inset shows the evolution of the normalized area under the peaks (*A*_*p*_/*A*_1_) with *p* (dots), and the lines are fits to illustrate the trends. (d) Peak separation as a function of *V*_bg_. The vertical error bars are the uncertainties from the *B*_f_ vs *p* linear fit, and the horizontal ones account for a 0.1 V uncertainty of the CNP. The black line is the result from eq 1 assuming normal incidence from the QPCs (θ = 0). The gray area corresponds to the experimental error from determining *n* (14%) and *L* (10%). Simulated TEF signal for perfectly aligned (e) and 3° misaligned (f) QPCs with respect to the armchair crystallographic direction. The black curves were obtained by adding the *K* and *K*′ valley-resolved spectra. The insets show the trigonally warped trajectories corresponding to the average incidence angles for valleys *K* and *K*′, and the dark yellow rectangles represent the gate-defined edges.

TEF measurements were performed using configuration C1, which is shown in Figure 2a. A current (*I*) is applied to the right QPC to generate electron flow into the ballistic BLG channel that is steered using the out-of-plane *B* field. To detect the ballistic skipping orbits, the nonlocal voltage (*V*_nl_) is measured between the left QPC and a reference electrode connected to the left of the BLG channel. To avoid voltage *V*_nl_ offsets, we used a differential DC measurement technique to obtain the TEF spectra in Figures 2 and 3.

**Figure 3:**
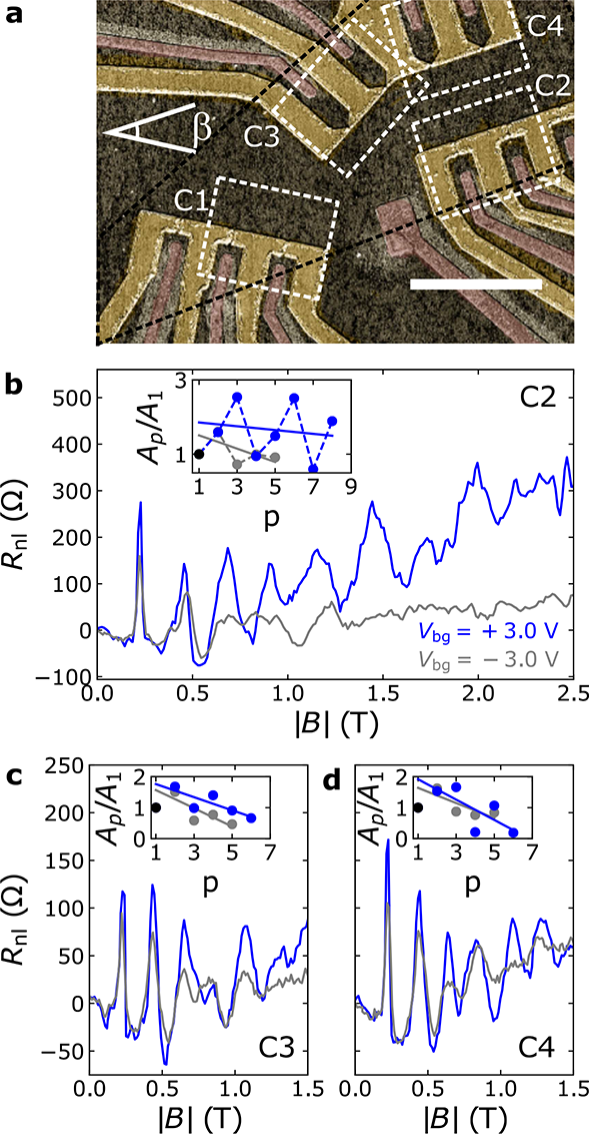
TEF along different crystallographic directions at 1.8 K. (a) False-color AFM image with the QPCs involved in C1 (Figure 2), C2, C3, and C4 delimited by white dashed lines. C2 is rotated an angle β = 30° with respect to C1 and C4 is rotated β = −30° with respect to C3. The scale bar is 5 μm. The BLG edges are indicated by black dashed lines. (b–d) TEF in configurations C2–C4 at *V*_bg_ = ±3 V. The insets show the normalized peak area vs *p* and the lines are linear fits to illustrate the trend.

The results from such measurements performed for different *V*_bg_ values are shown in Figure 2b. Note that, to ensure that the charge transport occurs only through the QPCs, we have adjusted *V*_tg_ to keep the double-gated regions charge neutral (diagonal line in Figure 1b). We first consider the *V*_bg_ = −4 V case. For *B* < 0, the signal is zero (dashed lines) or smaller than the noise level of the measurement, which is 2 Ω, consistent with the fact that the ballistic electron stream deviates toward the right and does not generate a signal on the detector. In contrast, when *B* > 0, five clear focusing peaks are observed, indicating that even though the QPC conductance is not quantized for *V*_bg_ < 0 (Figure 1c), the hole trajectories are well-defined and reflection at the gate-defined edge between both QPCs is smooth. As *V*_bg_ approaches zero, *n* in the BLG channel decreases, and the distance between the peaks becomes smaller. At *V*_bg_ > 0 peaks occur for *B* < 0, consistent with the fact that the carriers have changed from holes to electrons.^9,10,13^

For a more detailed comparison, in Figure 2c we show the *V*_bg_ = ±3 V spectra, where the bandgap is approximately 45 meV.^29^ Two clear differences can be distinguished: (i) The *p* = 1 peak is 2 times higher for *V*_bg_ = +3 V. This is most likely due to the lower *G* at *V*_bg_ = +3 V, which converts the collector current (*I*_c_) into the measured *V*_nl_ = *I*_c_/*G*. As shown in Figure 1c, *G* is roughly 2 times larger for *V*_bg_ = −3 V than for *V*_bg_ = +3 V, explaining most of the measured asymmetry in the *p* = 1 peak magnitude. (ii) The peak amplitude decays with *p* much faster at *V*_bg_ = −3 V. To quantify the TEF signal decay with *p* and correct for a small contact magnetoresistance (see SI Section S4), we calculated the area under the TEF peaks^10^ normalized by the two-terminal resistance (see SI Section S5b). The result is shown in the inset of Figure 2c with a linear fit excluding the *p* = 1 peak (which has the smallest area). The obtained peak areas are fairly constant from *p* = 2 up to *p* = 8 (including the *p* = 4 peak, which occurs between 0.75 and 1 T and is split in two), indicating specular reflection. In contrast, for *V*_bg_ = −3 V, the peak area decays with increasing *p*.

The faster peak decay for *V*_bg_ < 0 can be explained in terms of a change of the QPC width (*W*). The finite *W* of the detector poses an upper bound of the maximum number of peaks that can be measured. In particular, if *r*_c_ cos θ ≤ *W*/2, all electrons will enter the detector and extra peaks cannot be detected,^7^ leading to *B* ≤ 2 T for *W* = 200 nm, cos θ = 1, and a circular trajectory. In contrast, for *W* = 100 nm, we obtain *B* ≤ 3.9 T. As shown in Figure 1c, *G* is almost 8 times smaller for electrons than for holes, indicating that a significant electron–hole asymmetry in the QPC width is plausible. Additionally, decreasing the injector *W* is known to lead to electron jets with improved collimation.^21,58^ Because the focusing length of a trajectory depends on its injection angle, the differences between focusing lengths of different trajectories increase with *p*. Thus, a narrow angular distribution is expected to help maintain a constant peak amplitude, even after several edge reflections.

In Figure 2b, for *V*_bg_ = 1.5 V (red curve), additional oscillations similar to those in refs (7 and 25) can be observed on top of the focusing spectrum. The amplitude of these oscillations decreases with increasing *V*_bg_, a result that is consistent with quantum interference between the different electron paths contributing to the TEF signal because the Fermi wavelength increases with decreasing *n*. For completeness, *R*_nl_ near *V*_bg_ = 0 V is shown in SI section S7.

To gain more insight into the measured TEF spectra, we analyzed the positions of the focusing peaks (*B*_f_) as a function of *p*. In particular, we determined *B*_f_ and fit it to *B*_f_ = *B*_0_ + (d*B*/d*p*) × *p*, where *B*_0_ and d*B*/d*p* are constants accounting for the magnet remanence and the average spacing between the peaks, respectively. In Figure 2d we show |d*B*/d*p*| and compare it with the result from eq 1 for normal incidence (θ = 0). The similarity between both curves further confirms that our signal is due to TEF. Even though the small underestimation of *B*_f_ by eq 1 could be due to trigonal warping,^9^ the orientation dependence of *B*_f_ expected from ref (9) is not observed here (see SI Section S6), preventing a conclusive statement.

The results shown in Figure 2c at *V*_bg_ = +3 V show features resembling a beating pattern. In particular, all the peaks except *p* = 1 and 4 can be decomposed into two narrower peaks, and the latter, which has a dip where one would expect a peak, can be decomposed into three well-separated peaks. Additionally, the Fourier transform of the TEF spectrum (see SI Section S5b for details) also indicates the presence of a beating pattern, implying a periodic modulation. Even though there may be a combination of impurities or irregularities at the confinement potential that could explain this effect, there is a fundamental reason to expect such features in the TEF spectra. BLG is known for showing trigonal warping; i.e., its Fermi surface is not circular. In this case, the emission of electrons by the QPCs occurs in jets that depend on the crystallographic orientation of the QPCs on the BLG. If the QPCs are slightly misaligned with respect to a crystallographic direction, the valley-polarized jets will be emitted with slightly different |θ|, leading to two different *B*_f_ values for the peaks in valleys *K* and *K*′.^40^

Semiclassical calculations considering the effect of trigonal warping (see SI Section S9 for details) are shown in Figures 2e and 2f for the perfectly aligned and the small misalignment (0.05 rad ≈ 3°) cases, respectively. The trajectories are shown in the insets. In the latter, a beating pattern arises that is compatible with the measured data.

To show the robustness of the TEF measurements and explore the role of the BLG crystallographic orientation in the TEF spectra, we have patterned QPCs in different directions on the same BLG flake. The relative angle between the QPC sets is 30° to compare the armchair with the zigzag crystallographic directions. As shown in Figure 3a, C2 is aligned parallel to the longest BLG straight edge with an accuracy of ∼5°. Because we expect the flake edge to be aligned with a crystallographic direction with an uncertainty of a few degrees,^59,60^ we assume that the C2 QPCs are aligned to a crystallographic direction with an accuracy of less than 10°. Thus, the 30° rotated C1 QPCs, are expected to be along the other. We compare the TEF spectra in Figure 2c with the TEF spectra obtained using configurations C2, C3, and C4 from Figure 3a, shown in Figures 3b, 3c, and 3d, respectively. The results show several features: (i) The TEF peaks decay faster with *p* for holes than for electrons in all the geometries. (ii) For C3 and C4, which contain hornlike QPCs not showing size quantization (see SI Section S6 for details), the decay in peak amplitude for electrons is more pronounced than for C1 and C2 where *G* is quantized. As a consequence, six peaks can be distinguished instead of eight. (iii) The width of the *p* = 1 peak is significantly smaller than that of the *p* = 2 peak in all the configurations, for both electron and hole doping. Observations (i) and (ii) show a correlation between *G* and the TEF peak amplitude decay, further indicating that the QPC width plays a relevant role in the peak amplitude decrease.

Because the occurrence of a beating pattern is very sensitive to a tiny misalignment, the lack of a clear beating pattern in Figure 3b is not inconsistent with the Fermi surface being warped.

To characterize the scattering sources in BLG, we have measured *R*_nl_ vs *B* at different temperatures (*T*) at *V*_bg_ = ±3 V. At 2 K, the peak height is the highest, and as *T* increases, the background becomes more pronounced and the focusing signal gets smaller. Comparing the 2 K with the 10 K measurements, the 2 K spectra contain extra features at positive and negative *B*-fields. A fast decay when increasing *T* indicates that these features are likely due to quantum interference, as the phase-coherence length is known to drop within this range.^61^

To extract the *T* dependence of the scattering rate (τ_p_^–1^) from Figures 4a and 4b, we have followed ref (10) (see SI Section S5 for details). The result obtained using the area under the *p* = 2 peak is shown in Figure 4c. Here, the dots correspond to the values extracted from Figure 4a,b, and the lines are fits to τ_p_^–1^ = *aT*^2^ + *bT* + *c*. Assuming a hard-wall confinement potential, a quadratic *T* dependence of τ_p_^–1^ is associated with electron–electron interactions.^10,62,63^ In contrast, a linear dependence is associated with phonon-dominated scattering.^9,64^ The fits indicate that electron–electron interactions may play a relevant role in the *T*-dependent scattering for electrons, but not for holes; see SI Section S5 for the fitting parameters and a more detailed discussion. We suspect that the background signals in Figure 4 are caused by a small miscalibration of *V*_tg_.

**Figure 4:**
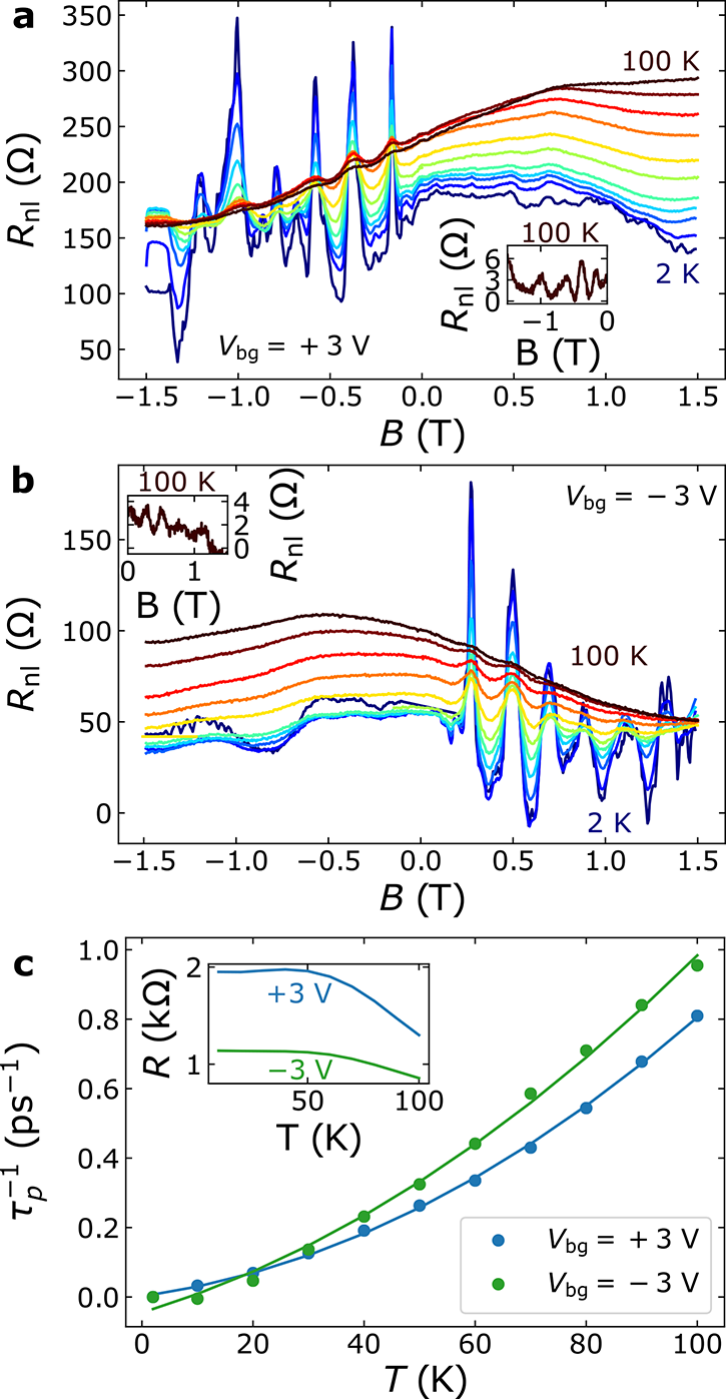
Temperature dependence of TEF in BLG. *B* dependence of *R*_nl_ for *T* = 2, 10, 20, ..., 100 K at (a) *V*_bg_ = +3 V and (b) *V*_bg_ = −3 V. The insets correspond to the 100 K data with a smooth background corrected. (c) Scattering rate estimated using the spectra in panels a and b (dots) and its fit to a parabola (lines). The inset shows the *T* dependence of the QPC resistance.

To conclude, we have measured the TEF in hBN-encapsulated BLG devices where QPCs are defined in different directions using electrostatic gating. Our results show eight focusing peaks with similar amplitudes together with quantum interference features. By comparing TEF spectra with semiclassic simulations, we identify a periodic modulation of the peak size that is consistent with the effect of trigonal warping. Moreover, the TEF temperature dependence shows that the signal persists up to 100 K. Our results are promising for future valleytronic devices.

## Data Availability

All the data and code associated with the analysis and theoretical simulations are available free of charge from ref (65).

## Supporting Information Available

Details on the fabrication and measurement techniques, characterization of the backgate capacitance, electronic mobility, two-terminal magnetoresistance, extraction of the area under the focusing peaks, the complete TEF data set, the results obtained in sample 2, and numerical simulations (PDF)
