# Supplementary material for: Specular Electron Focusing between Gate-Defined Quantum Point Contacts in Bilayer Graphene
Source: Nano Lett. 2023 Jun 8;23(12):5453–9. doi: 10.1021/acs.nanolett.3c00499 (PMC10311585; doi:10.1021/acs.nanolett.3c00499)
Supplement: Supplementary file 1 — nl3c00499_si_001.pdf [file nl3c00499_si_001.pdf]

Supporting information of "Specular electron focusing between gate-defined quantum point contacts in bilayer graphene"

Josep Ingla-Aynés,[1,*] Antonio L. R. Manesco,[1] Talieh S. Ghiasi,[1] Serhii Volosheniuk,[1] Kenji Watanabe,[2] Takashi Taniguchi,[3] and Herre S. J. van der Zant[1]

[1]*Kavli Institute of Nanoscience, Delft University of Technology, Lorentzweg 1, 2628 CJ Delft, The Netherlands*

[2]*Research Center for Functional Materials, National Institute for Materials Science, 1-1 Namiki, Tsukuba 305-0044, Japan*

[3]*International Center for Materials Nanoarchitectonics, National Institute for Materials Science, 1-1 Namiki, Tsukuba 305-0044, Japan*

(Dated: June 2, 2023)

CONTENTS

S1. Methods 2
A. Device fabrication 2
B. Measurement techniques 4

S2. Backgate capacitance obtained using Shubnikov–de Haas oscillations 4

S3. Electronic mobility 5

S4. Two-terminal magnetoresistance at different temperatures 7

S5. Area under peaks 8
A. Different temperatures 8
1. Effect of the R_c-dependence with temperature 11
B. Base temperature 12

S6. Transverse electron focusing at different geometries 14
A. Reciprocity 18

S7. Transverse electron focusing near the charge neutrality point 19

S8. Transverse electron focusing Sample 2 20

S9. Numerical simulations 22
A. Tight-binding model 22
B. Semiclassical calculations 23

References 25

S1. METHODS

A. Device fabrication

Samples have been prepared using the poly (bisphenol A) carbonate (PC) technique [1, 2]. The hexagonal boron nitride (hBN) and bilayer graphene (BLG) flakes were exfoliated from

* J.InglaAynes@tudelft.nl

bulk crystals [3] and picked up with a PC layer at temperatures between 60 and 90 °C. The resulting heterostructure was released on a clean SiO_2 substrate with Au markers by melting the PC layer at temperatures above 150 °C. Subsequently, the PC covering the stack was removed from the surface by dissolving it in chloroform. The stack was then annealed for 1 h in an Ar atmosphere at 400 °C before contact preparation. At this stage, an atomic force microscopy (AFM) image was taken in the AC mode of a Cypher AFM to determine the thickness of the different flakes (Fig. S1a). The profiles extracted to obtain the thickness of the hBN flakes are indicated by a green and a blue line and shown in Fig. S1b, together with the estimated flake thicknesses. The bottom hBN thickness with respect to the SiO_2 (left step green profile) is 26 nm, 6.5 nm larger than the 19.5 nm obtained when the bottom hBN is covered by the top hBN (blue profile). On the one hand, this discrepancy may be due to the different adhesion of SiO_2 and hBN surfaces, affecting the left step of the green profile. On the other hand, the slight negative slope of the lower plateau of the blue line, combined with its smoother profile (caused by the top hBN coverage) may lead to a thickness underestimation. We take the average (23 nm) as the estimate of the bottom hBN thickness.

The contacts to the bilayer graphene were defined using e-beam lithography. After defining the contact pattern, the top hBN was etched with a mixture of CHF_3 and O_2 with a 10 to 1 flow ratio, 40 W of power and a pressure of 5 μbar [4]. This recipe gives an etch rate for hBN of approximately 30 nm/min and leaves the BLG edges exposed. After etching, the Ti(5 nm)/Au(35 nm) electrodes were deposited using e-beam evaporation. The top gates were prepared using the same method replacing the CHF_3/O_2 etching by a mild O_2 etching (10 s 15 W) to promote adhesion between the Ti and the hBN surfaces. The final result is shown in Fig. S1c: Rectangle A surrounds the contacts involved in C1, rectangle B the contacts in C2 and C2L, and rectangle C, the contacts in C3 and C4 (see Section S6). Note, that Fig. S1c corresponds to the false-colored phase channel of an AFM image. The contacts to the BLG (dark dashed line) are red and the top gates (yellow) surround the contacts to create the electrostatically-defined quantum point contacts (QPCs). There is one extra contact above and another one below the AFM image which were used as reference for the nonlocal measurements.

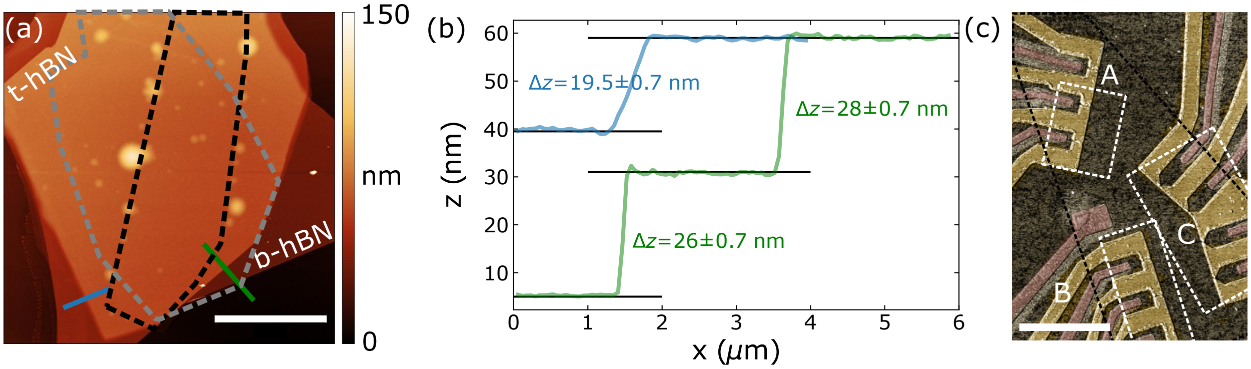

Figure S1. (a) AFM image of the device after annealing. The black dashed edge corresponds to the BLG, the gray edge to the multilayer graphene backgate and t(b)-hBN to the top (bottom) hBN flakes. The scale bar is 10 μm. (b) Height profiles extracted along the blue and green lines in panel a with the extracted step heights corresponding to the hBN thicknesses. The error range accounts for the roughness of the hBN surfaces (0.5 nm). (c) Phase of an AFM image of the completed device with the BLG edges in black. The dark yellow Ti/Au structures are top gates used to define QPCs and the brown ones are contacts to the BLG. The scale bar is 5 μm.

B. Measurement techniques

The measurements shown in Figs. 1, 2 and 3 of the main manuscript and Figs. S7 and S8 are performed using a differential DC measurement technique under an applied current $I = 100$ nA. In each point the current is applied following the sequence: $I_1 = +I$, $I_2 = -I$, $I_3 = +I$ while the nonlocal voltage is measured after waiting 100 ms at each stage. The nonlocal voltages used here are obtained using $V_{\text{anti}} = ((V_1+V_3)/2-V_2)/2$, where $V(I_{1,2,3}) = V_{1,2,3}$. This technique allows us to correct for any background signals. For completeness, $V_{\text{sym}} = ((V_1+V_3)/2+V_2)/2$ is also extracted from the measured data and can be retrieved from [5]. The TEF measurements at different T were performed using a standard lock-in technique at a frequency $f \approx 18$ Hz.

S2. BACKGATE CAPACITANCE OBTAINED USING SHUBNIKOV–DE HAAS OSCILLATIONS

To determine the capacitance of the backgate (C_{bg}) in an accurate way we have used Shubnikov-de Haas oscillations. In a four-terminal measurement configuration, we measured

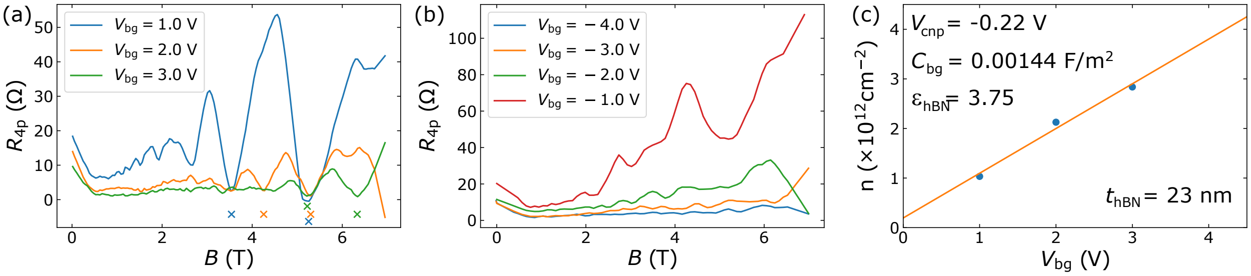

Figure S2. (a) and (b) R_{4p} vs. B up to 7 T for positive and negative $V_{\rm bg}$, respectively. The crosses in panel a indicate the B-values used to obtain the carrier density. (c) Carrier density extracted from panel a at $V_{\rm bg}$=1, 2 and 3 V with the linear fit to the n vs. $V_{\rm bg}$ dependence used to extract the backgate capacitance.

the longitudinal four-point resistance (R_{4p}) at different $V_{\rm bg}$ while sweeping B up to 7 T. The results from such a measurement are shown in Figs. S2a and S2b for positive and negative $V_{\rm bg}$, respectively. Since for negative $V_{\rm bg}$ the measured data does not show clear oscillations to extract the carrier density (n), we have only used positive $V_{\rm bg}$. To determine n, we have used [3]:

$$n = \frac{4e}{h}\frac{B_{i+1}B_i}{B_{i+1}-B_i} \tag{S1}$$

where e is the electron charge, h is the Plank constant and B_i and B_{i+1} correspond to the adjacent field positions where R_{4p} is minimal, indicated as crosses in Fig. S2a. The result from Equation S1 is shown in Fig. S2c and plotted vs. $V_{\rm bg}$ as blue dots. Because the carrier density changes with the gate voltage following $n = C_{\rm bg}(V_{\rm bg} - V_{\rm cnp})/e$, where $V_{\rm cnp}$ is the position of the charge neutrality point, we have used a linear fit $n = AV_{\rm bg} + B$ (orange line) to extract $C_{\rm bg}$= Ae and $V_{\rm cnp} = -B/A$. With $\epsilon_{\rm hBN} = C_{\rm bg}t_{\rm hBN}/\epsilon_0$, where ϵ_0 is the vacuum permittivity, and the bottom hBN thickness from AFM measurements ($t_{\rm hBN} \approx 23$ nm), we estimate its dielectric constant, $\epsilon_{\rm hBN} \approx 3.75$, in agreement with [6].

S3. ELECTRONIC MOBILITY

To estimate the device mobility, we have measured the BLG channel resistance as a function of $V_{\rm bg}$. The measurement geometry is shown in Fig. S3a and the result for the square resistance $R_{\rm sq}$= $V \times W/(I \times L)$ is shown in Fig. S3b. Here, $W = 7.2\,\mu$m is the BLG width between the V probes and $L = 2\,\mu$m is the separation between them. In addition

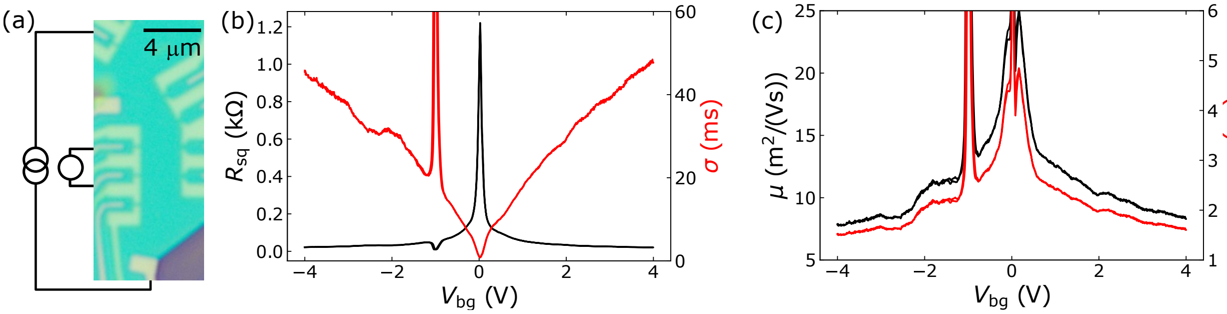

Figure S3. (a) Optical microscope image of Sample 1 with the measurement circuit. (b) R_{sq} and $\sigma = {R_{\text{sq}}}^{-1}$ vs. V_{bg}. (c) Mobility and momentum scattering time vs. V_{bg}.

to the expected trend with a peak at the charge neutrality point (CNP) [3], we observe a dip at $V_{\text{bg}}= -1$ V. From the two-point measurements used to characterize the QPCs (which show a peak for $V_{\text{bg}}\approx -1$ V), we conclude that it corresponds to the CNP of the BLG near the contacts. Thus, we attribute the drop in the measured resistance to the gate-tunable invasiveness of the voltage probes, which is minimal for $V_{\text{bg}}\approx -1$ V, when the contact resistance is maximal, resulting in an enhancement of the effective mobility. This explanation is consistent with the channel being ballistic and the contacts having a small overlap with the current path.

We have also plotted $\sigma = {R_{\text{sq}}}^{-1}$ vs. V_{bg}, which does not show the linear trend with V_{bg} as expected from the Drude model for constant mobility (μ), that predicts $\sigma = ne\mu$. This shows that the effective μ depends on n. To determine μ (Fig. S3c) we have used $\mu = \sigma/(ne)$ and used μ to estimate the momentum scattering time $\tau_p = m^*\mu/e$, where $m^* = 0.034 \times m_e$ is the effective mass in BLG [7] and m_e the electron mass. With $v_f \approx 0.5 \times 10^6$ m/s, the mean-free-path $l_{\text{mfp}} = v_f \times \tau_p \sim 1\,\mu$m, is comparable to the 2 μm separation between the V probes. This observation indicates that the channel is in the ballistic regime and the measured values represent a lower bound to the actual device quality. The underestimation of τ_p using this method is confirmed by the clear observation of focusing between QPCs placed at a distance of $L = 4\,\mu$m, requiring a ballistic path of $L \times \pi \approx 12.6\,\mu$m. Finally, there is not a significant difference between the electron and hole mobilities for $|V_{\text{bg}}| > 1$ V, indicating that the electron-hole asymmetry in the focusing signals is not caused by a difference in mobility.

S4. TWO-TERMINAL MAGNETORESISTANCE AT DIFFERENT TEMPERATURES

To characterize the QPCs, we have measured the B-dependence of the two-terminal resistance at $V_{\mathrm{bg}}= \pm 3$ V at different T. The result is shown in Figs. S4a and S4b and shows small oscillations at low T. There are two main features to highlight from these figures: Firstly, the collector resistance (R_{c}) decreases with increasing temperature. This decrease is caused by the small bandgap opening at the BLG under the split gates (around 45 meV for an applied electric field of approximately 0.5 V/nm [8]), that exhibits thermally activated behavior and has to be taken into account to analyze the T-dependence of the focusing signal. Secondly, the magnetoresistance below 0.5 T is smaller than 10% in both cases, allowing us to analyze the low-B focusing peaks assuming that R_{c} is constant through the B-sweep.

For $V_{\mathrm{bg}}= -3$ V, the low-T data shows a small peak at $B = 0$, that is absent for $V_{\mathrm{bg}}= +3$ V. This peak resembles weak localization (WL). Its large width indicates that, if its origin is WL, it must come from a region with a small phase coherence length (of the 100 nm range). We conclude that it most likely originates from WL near the contacts between the BLG and Ti/Au electrodes, which have been doped by the etching process and are expected to have worse transport properties than the rest of the channel.

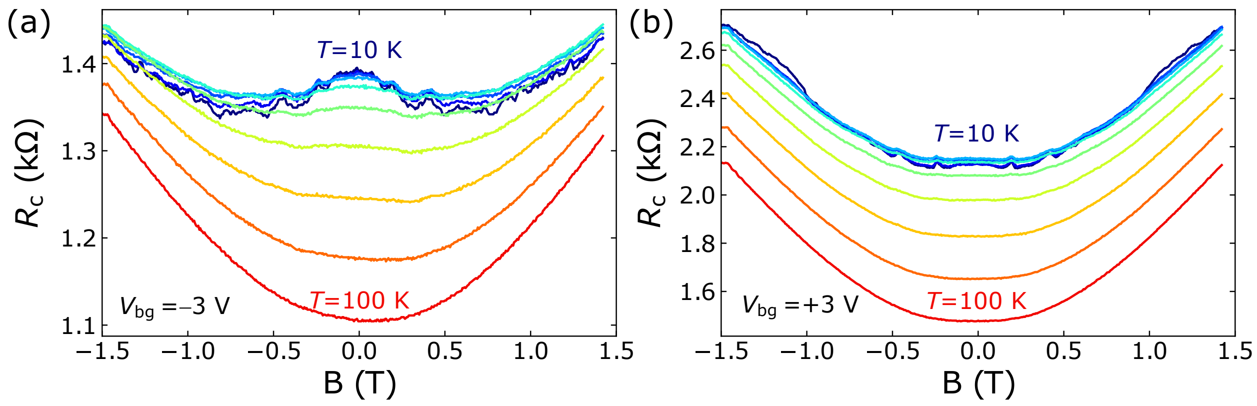

Figure S4. Quantum point contact magnetoresistance at V_{bg}−3 V (a) and $V_{\mathrm{bg}}= +3$ V (b).

S5. AREA UNDER PEAKS

A. Different temperatures

To determine how the scattering rate changes with T we have extracted the area under the focusing peaks shown in Fig. 4 of the main manuscript. This operation requires the assumption that the reference electrode, which is placed far from the injector, only contributes to a smooth background in the measured signal. Such assumption is justified considering the long distance between this electrode and the injector and the large effective width it has due to the absence of a pair of confining top gates.

The area under the peaks has been extracted following several steps:

- $R_{\rm nl}$ has been normalized by $R_{\rm c}$.
- A linear background has been corrected from the data.
- The data has been slightly smoothed (see the small difference between the black curves and colored scatters in Fig. S5a-d).
- The minima in the nonlocal signal have been identified using the find_peaks function from the python package scipy.signal on the reversed data (-$R_{\rm nl}/R_{\rm c}$).
- The background has been defined for each peak by linear interpolation between the extreme points.
- The area between the data and background has been calculated using the trapz function from the python package numpy.
- The scattering rate τ_p^{-1} has been calculated using [9]:

$$\tau_p^{-1} = -2v_{\rm F}/(\pi L)\log(A_p(T)/A_p(T_{\rm base})), \tag{S2}$$

where $v_{\rm F} \approx 9.0 \times 10^6$ m/s is the Fermi velocity of BLG at $V_{\rm bg}$= +3 V, $A_p(T)$ is the area under the p-focusing peak (starting from $B = 0$) at each T and $A_p(T_{\rm base})$ is the area of this peak at $T = 2$ K.

The result of the process described above is shown in Fig. S5a-d at $V_{\rm bg}$= $+(-)3$ V and T = 50, 30 and 20 K, respectively. The T values have been chosen to illustrate the

assimilation of the low-B feature below the $p = 1$ peak that affects the T dependence of τ_p^{-1}. Figs. S5e-S5h show that the $p = 1$ and $p = 2$ dots overlap for $T \leq 40(20)$ K for $V_{\rm bg}$= +(−)3 V. At higher T, the structure shown in Fig S5a-S5d is assimilated by the $p = 1$ peak, leading to an increase of the peak area which causes a spurious decrease of τ_p^{-1}. For this reason, we have used the $p = 2$ result for our analysis.

In Figs. S5e-S5h we have fit τ_p^{-1} vs T to two parabolas: $\tau_p^{-1} = aT^2 + bT + c$ (Figs. S5e and S5f), and $\tau_p^{-1} = aT^2$ (Figs. S5g and S5h). The former fits the $p = 2$ result better for both $V_{\rm bg} = +3$ V and -3 V.

A quadratic T-dependence of τ_p^{-1} is associated with electron-electron interactions [9]. In contrast, a linear dependence is associated with phonon-dominated scattering [10, 11]. Thus, our analysis indicates that both scattering terms are relevant. By calculating $T_0 = b/a$, which is the T where the quadratic term starts to dominate over the linear term, we obtain $T_0 = 37 \pm 3$ (90 ± 20) K for $V_{\rm bg}$= +(−)3 V.

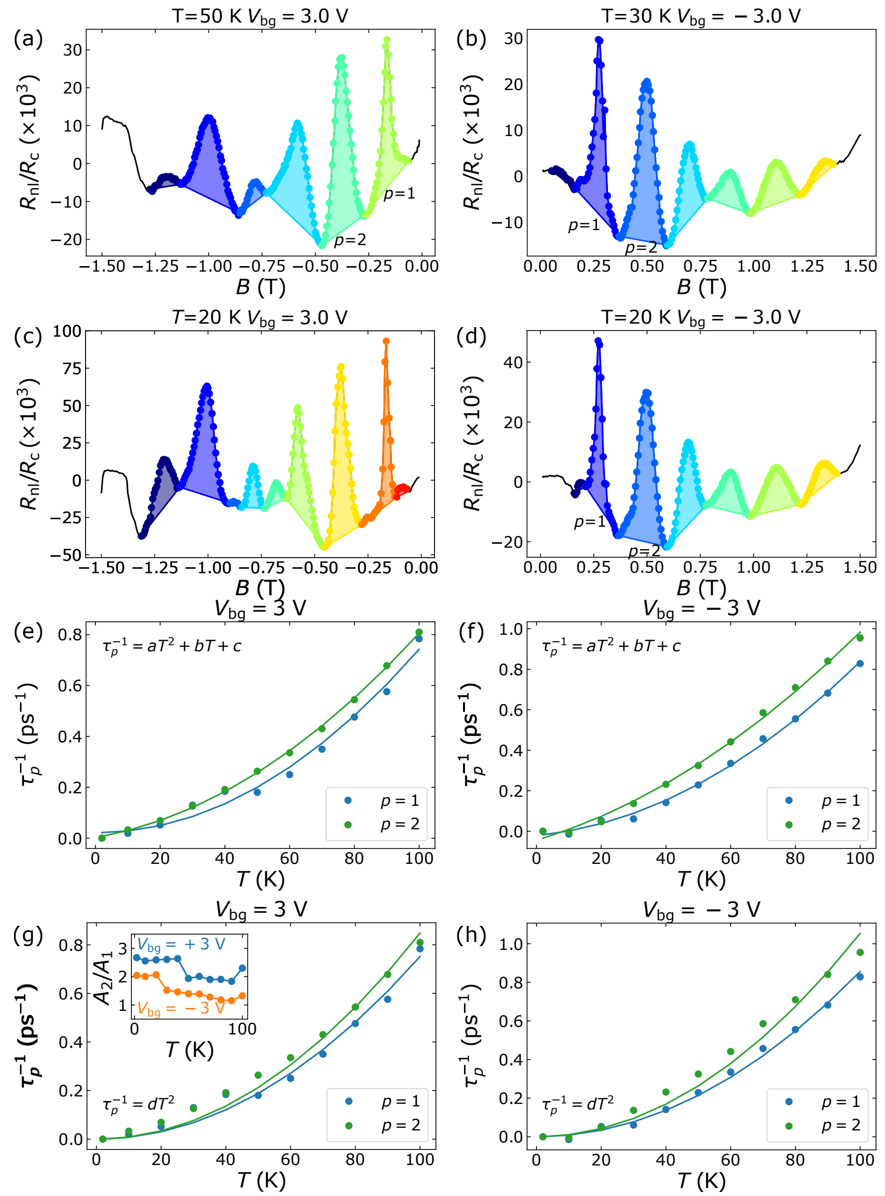

Figure S5. (a)-(d) Focusing spectra with the area under the peaks colored. The scatter plot shows the processed data (see text) while the dark line at the background the raw measurement. (e)-(h) Scattering rate τ_p^{-1} as a function of T from the first two focusing peaks at $V_{\rm bg}= \pm 3$ V. The $p=1$ peak merges with a smaller structure at $T=50(30)$ K for $V_{\rm bg}=+3(-3)$ V, leading to a jump in τ_p^{-1} extracted from this peak. In panels (e) and (f) τ_p^{-1} vs. T is fit to aT^2+bT+c, whereas in (g) and (h) it is fit to dT^2. The fitting parameters can be found in Table S1. The inset of panel b shows the ratio A_2/A_1 vs. T at $V_{\rm bg}= \pm 3$ V.

TABLE S1. Fitting parameters obtained from the temperature dependence of τ_p using $\tau_p^{-1} = aT^2 + bT + c$ (Figs. S5e and S5f) and $\tau_p^{-1} = dT^2$ (Figs. S5g and S5h).

		a (ps^{-1}K^{-2})	b (ps^{-1}K^{-1})	c (ps^{-1})	d (ps^{-1}K^{-2})
V_{bg}= +3 V	$p = 1$	$(7 \pm 1) \times 10^{-5}$	$(-0.09 \pm 1) \times 10^{-3}$	$(2 \pm 3) \times 10^{-2}$	$(7.5 \pm 0.2) \times 10^{-5}$
	$p = 2$	$(5.9 \pm 0.3) \times 10^{-5}$	$(2.2 \pm 0.3) \times 10^{-3}$	$(3 \pm 6) \times 10^{-3}$	$(8.5 \pm 0.2) \times 10^{-5}$
V_{bg}= −3 V	$p = 1$	$(7.1 \pm 0.6) \times 10^{-5}$	$(1.5 \pm 0.7) \times 10^{-3}$	$(-4 \pm 2) \times 10^{-2}$	$(8.6 \pm 0.1) \times 10^{-5}$
	$p = 2$	$(5.5 \pm 0.8) \times 10^{-5}$	$(4.8 \pm 0.9) \times 10^{-3}$	$(-4 \pm 2) \times 10^{-2}$	$(1.05 \pm 0.03) \times 10^{-4}$

Since we are analyzing the $p = 2$ peaks, it would be tempting to attribute the electron-hole asymmetry to diffuse scattering at the edge (DSE), that the TEF spectra indicates may be stronger for holes. If DSE was T-dependent, it could lead to a faster $p = 2$ peak decay with T and artificially enhance t_p^{-1}. By monitoring the T-dependence of A_2/A_1 we can determine whether the bT term is dominated by T-dependent DSE because, in this case, A_2/A_1 would decrease linearly with increasing T. We have plotted A_2/A_1 at the inset of Fig. S5b and found that the most clear feature is a sudden drop at $T = 50$ (30) K for V_{bg}= +3 (−3) V, as expected from Fig. S5, indicating that the dominant scattering source giving rise to the TEF amplitude decay with T at V_{bg}= +3 V and the linear T-dependence of τ_p^{-1} is not DSE. Note that the 100 K case shows a clear difference with respect to the others. We attribute it to the thermally-activated transport across the weakly-gaped ($\sim$ 40 meV) BLG region at 100 K where $k_B T \approx 8.6$ meV.

1. *Effect of the R_{c}-dependence with temperature*

The analysis shown above, which assumes that the $\tau_p^{-1} = aT^2$ term is caused by electron-electron interactions, is based on the results from Ref. [9], where the following result is obtained for monolayer graphene and a hard wall potential:

$$\tau_{ee}^{-1} \approx \frac{\alpha (k_B T)^2}{v k_F} \log\left(\frac{\beta \pi L}{2A}\right), \tag{S3}$$

where $\alpha \approx 0.518$, $\beta \approx 2.28$, $v = 1 \times 10^6$ m/s is the Fermi velocity of graphene, k_B is the Boltzman constant, k_F is the Fermi wavevector, and $A = \sqrt{w_c^2 + w_i^2}$, where $w_{(i)c}$ is the (injector) collector QPC width that determines the electron collimation. This implies that

a change of the widths with increasing T could affect our analysis. To quantify this effect, we have assumed that the changes in R_c with T shown in Fig. S4 are due to the activation of the BLG regions near the QPC which have a reduced bandgap. Under this assumption, the changes in R_c should correspond to changes in $w = w_i \approx w_c$ following $R_c \propto 1/w$. If we assume that w = 50 nm, the separation between the split gates, and take an upper bound to the change in QPC resistance with T of 50%, we obtain a correction to the log in Equation S3 of 18% at 100 K. Because w is used to determine the electron jet collimation [9] and electron jets emitted by QPC have been shown to be collimated [12], this correction is expected to be an upper bound. However, to make our analysis more robust against this effect, we have performed fits to $\tau_p^{-1} = aT^2 + bT + c$ for V_{bg}= +3 V limiting the T range to 80 and 60 K, where the correction is expected to be of less than 5%. In the former case, $T_0 = 50 \pm 10$ K has been obtained and, in the latter, $T_0 = 70 \pm 10$ K. The fact that both values are below 100 K indicates that the τ_{ee}^{-1} term dominates over the linear term at 100 K, consistently with Ref. [13].

B. Base temperature

To determine the specularity of the edge reflection in the TEF measurements, we have determined the area under the different peaks in the R_{nl} data shown in Fig. 2c of the main manuscript. To take into account the contact magnetoresistance shown in Fig. S4 we have normalized R_{nl} by R_c using the data from Fig. S4 at T = 10 K. The result, obtained following the procedure described in Section S5 A, is shown in Fig. S6 and shows that, for V_{bg}= −3 V, the peak amplitude decays much faster than for V_{bg}= +3 V, as shown in the main manuscript. Additionally, the amplitude of the V_{bg}= +3 V signal is still around 25% larger than the V_{bg}= −3 V case. We attribute this small difference to the fact that the detector has a slightly larger asymmetry at V_{bg}= ±3 V than the injector, which is the contact we corrected for. At the inset of Fig. S6b we show the normalized area under the different peaks in both V_{bg}= −3 V and V_{bg}= +3 V cases where one can see more clearly the faster decrease of peak amplitude in the former.

An additional feature which can be identified in the V_{bg}= +3 V data is the apparent beating pattern which results in the splitting of the $p = 4$ TEF peak. To infer whether it is compatible with the expected interference pattern arising from TEF between QPCs which

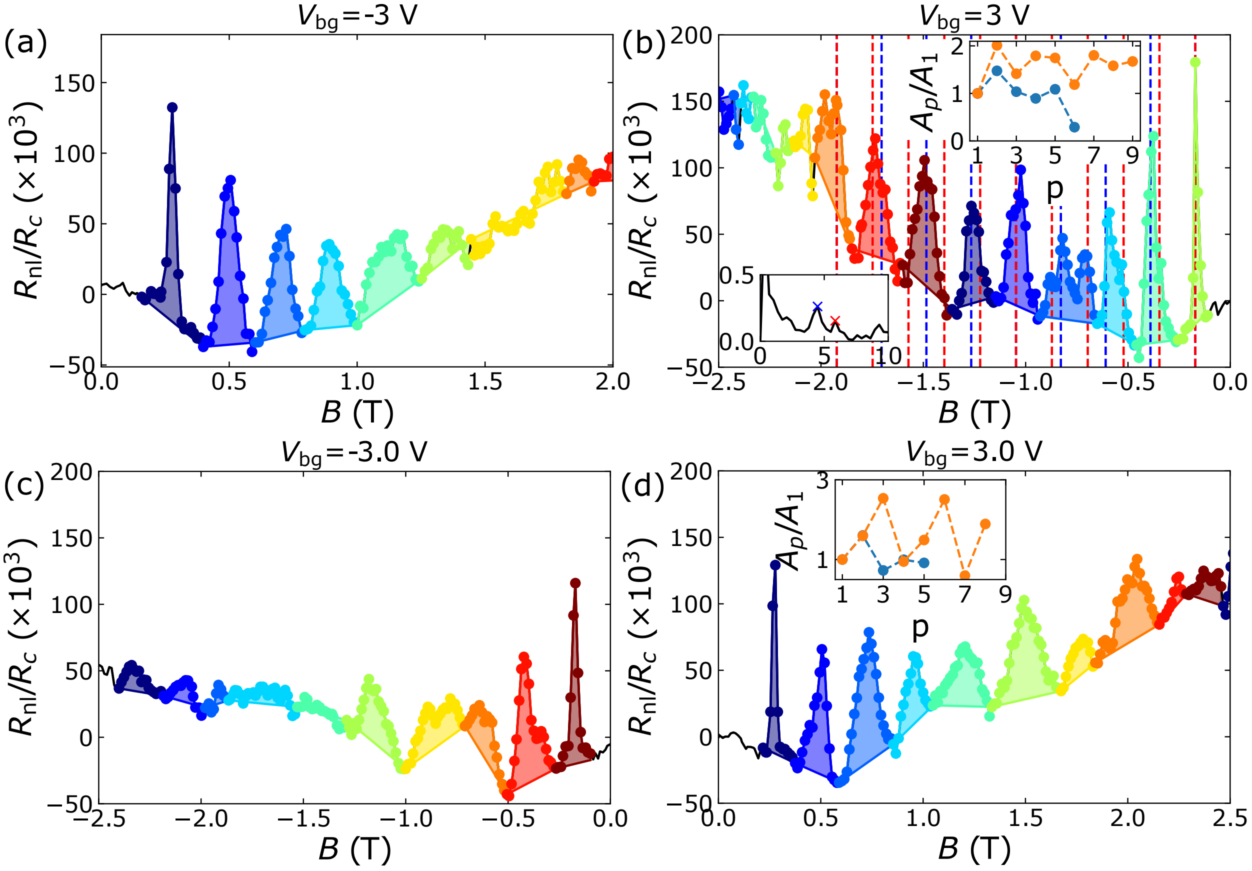

Figure S6. Focusing spectra obtained in configuration C1 and in C2 at V_{bg}= +3 V (a), (c) and V_{bg}= −3 V (b), (d), respectively. The area under the peaks is colored and the scatter plot shows the processed data while the dark line at the background the raw measurement (see text). The upper insets in panels b and d show the area under peak p (A_p) normalized by the area under the first peak (A_1) for V_{bg}= −3 V (orange) and V_{bg}= +3 V (blue). The equally-spaced blue and red vertical lines in panel b indicate the expected peak positions according to the Fourier analysis, which is shown at the lower inset of panel b. The y-axis is the amplitude of the Fourier transform of the TEF spectrum and the x-axis the Fourier frequency. The selected peaks are marked by crosses and color-coded according to the vertical lines.

are slightly misaligned with respect to a crystallographic direction, we calculated the Fourier transform of the signal. The result, shown at the lower inset of Fig. S6b, indicates that two clear peaks are present at the expected FFT frequency range. Because the measured B fields are not exactly equally spaced, the measured data has been mapped on a B axis with equally spaced points by interpolation from the raw TEF data. Because the TEF peaks are narrow,

details on the mapping such as small offsets in B can modify the FFT peak shapes. To correct for this issue we used a mesh of 1k equally spaced points from 0.1 to 3 T. As a result the FFT peak positions are robust against B shifts up to 0.1 T. The peaks obtained at the expected frequencies are marked by a red and a blue cross and their frequencies are inverted to determine the corresponding periodicities, which are represented as vertical dashed lines. The result, which is compatible with the first six peaks, indicates that a beating pattern with two separate frequencies can explain most of the spectra obtained at $V_{\mathrm{bg}}= +3$ V.

S6. TRANSVERSE ELECTRON FOCUSING AT DIFFERENT GEOMETRIES

In the presence of trigonal warping, the transverse electron focusing (TEF) spectra are expected to depend on the relative orientation of the QPCs with respect to the crystallographic directions of the BLG [14, 15]. For this reason, we have studied TEF using gate-defined QPCs which are oriented in different directions. In particular, the geometry used to obtain the TEF spectra shown in the main manuscript, which we also show in Fig. S7d, has a rotation of 30° with respect to the second set of QPCs, which is shown in Fig. S7e (see Fig. S1c for an overview of the whole sample). Since the QPCs in area B (Fig. S1c) are oriented along a straight edge in the BLG, which is most likely a crystallographic direction [16], the QPCs in area A are expected to be aligned along an armchair (or zig-zag) direction while the ones in area B must be along a zig-zag (armchair) direction, although we cannot tell which one is which.

Comparing Fig. S7d with Fig. S7e, the spectra occur at opposite B. This happens because in Fig. S7d the current source is at the left of the voltage probe whereas in Fig. S7e it is at the right. For negative V_{bg} in Fig. S7e there are fewer peaks than in Fig. S7d and the $p = 2$ peak is split into two.

To find if the focusing peaks occur at the expected B, we use [17]:

$$B_{\mathrm{f}} = \frac{2p\hbar k_F \cos\theta}{eL} = \frac{2p\hbar\sqrt{n\pi}\cos\theta}{eL}, \tag{S4}$$

where $\hbar$ is the reduced Plank constant, k_f the Fermi wavevector, θ the electron incidence angle from the QPC with respect to the normal, and L the contact separation. Note that Equation S4 corresponds to Equation 1 of the main manuscript.

To compare Equation S4 with the measured data, we have identified B_{f} as the B values

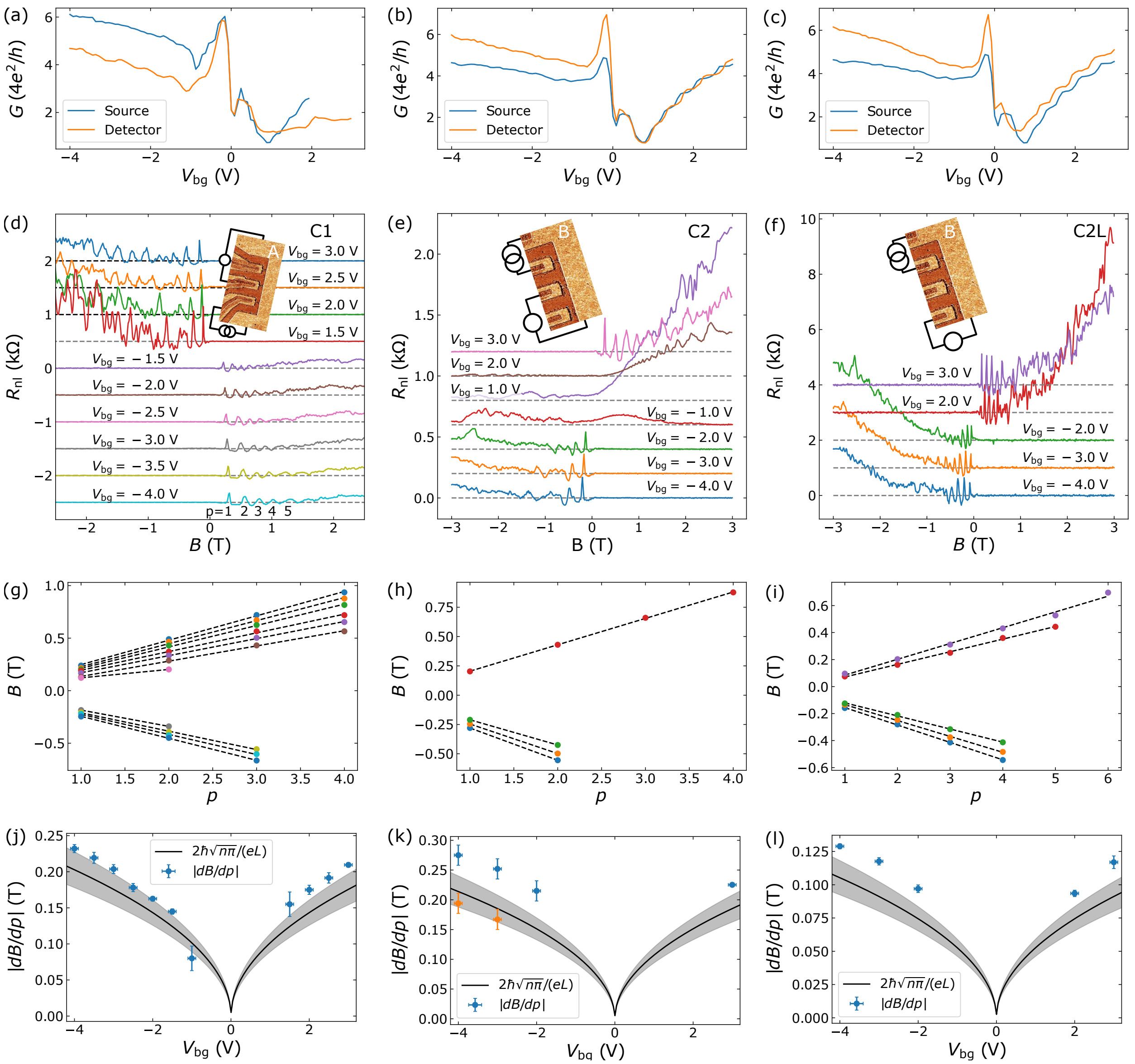

Figure S7. TEF measurements obtained using the QPCs in rectangles A and B of Fig. S1c. (a-c) G vs. V_{bg} calculated as in the main manuscript for the QPCs used in the TEF measurements shown in (d-f), respectively. The insets correspond to the measurement geometries represented with the same orientation as in Fig. S1c for clarity. (g-i) Peak positions vs. p at different V_{bg}. The dashed lines are the fits to $B = B_0 + (dB/dp) \times p$, where B_0 accounts for the magnet remanence and dB/dp is the slope. (j-l) Slope of fits obtained from panels g-i, respectively, together with a fit to Equation S4 assuming normal incidence ($\theta = 0$; dashed lines).

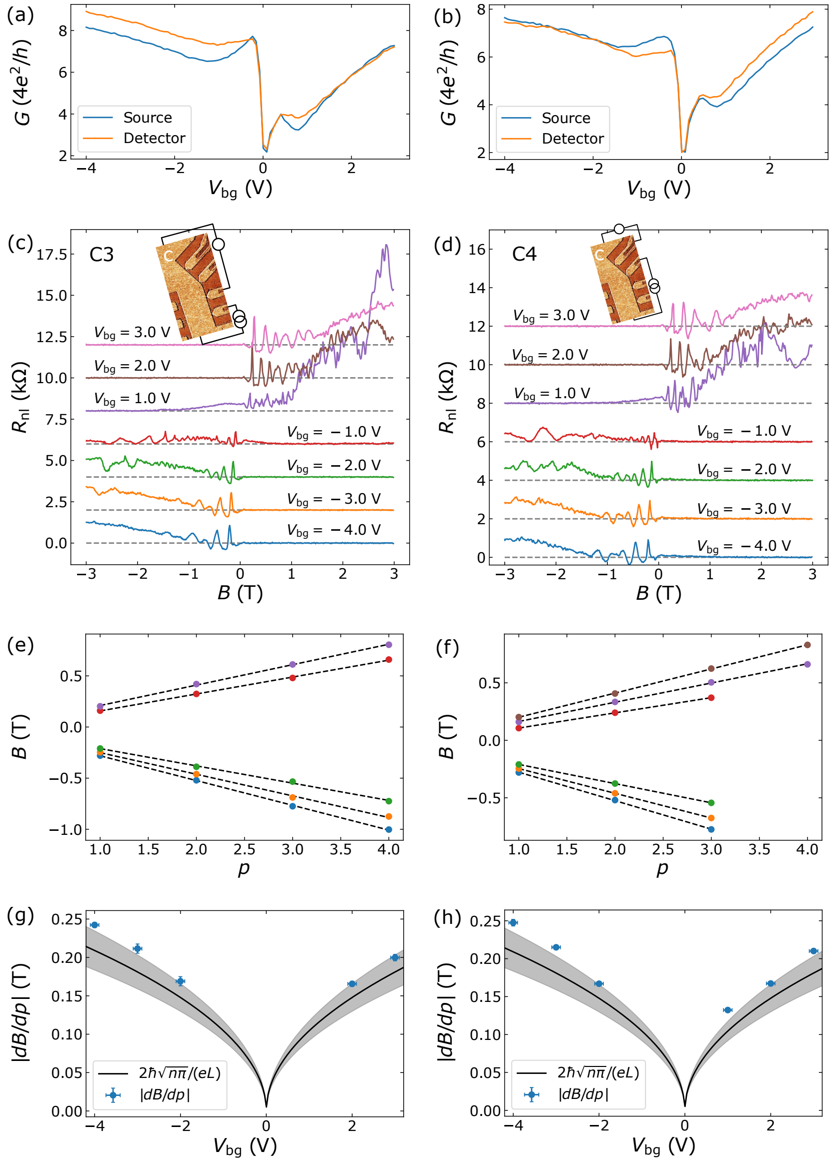

Figure S8. TEF measurements obtained using the QPCs in rectangle C of Fig. S1c. (a) and (b) G vs. V_{bg} calculated as in the main manuscript for the QPCs used in the TEF measurements shown in (c) and (d), respectively. The insets correspond to the measurement geometries represented with the same orientation as in Fig. S1c for clarity. (e) and (f) Peak positions vs. p at different V_{bg}. The dashed lines are fits to to $B = B_0 + (dB/dp) \times p$. (g) and (h) Slope of the fits obtained from panels e and f, respectively, together with a fit to Equation S4 assuming normal incidence ($\theta = 0$; dashed lines).

where R_{nl} is maximal, plotted B_{f} vs. p at each V_{bg}, and fitted the data to $B_{\mathrm{f}} = B_0 + (dB/dp) \times p$, where B_0 accounts for the coercivity of the magnet and dB/dp quantifies the change in B_{f} with p (see Figs. S7g-i). After this, we have plotted $|dB/dp|$ vs. n and compared this result with Equation S4 assuming $\cos(\theta) = 1$, which provides the maximal peak separation (see Figs. S7j-l). We observe that the separation between the measured peaks is even larger than predicted by Equation S4. This result is more pronounced in Fig. S7k, and may be consistent with an impurity obstructing the charge transport path and leaving a smaller peak at $B < B_{\mathrm{f}}$ (which we did not consider in Fig. S7h) and a larger peak at $B > B_{\mathrm{f}}$. As a result the spacing between the first and second peaks is artificially enhanced and, since the $p = 3$ peak is not clear enough, $|dB/dp|$ is overestimated. The opposite occurs when we consider the smaller peaks at lower B (Fig. S7k, orange dots).

For $V_{\mathrm{bg}}> 0$ the background signals in Fig. S7e are much larger than in Fig. S7d and additional features that are not expected from TEF are observed. We attribute them to quantum interference and restrict our comparison to $V_{\mathrm{bg}}= 3$ V. In this case, up to eight TEF peaks are visible, as in Fig. S7d, but without the splitting of the $p = 4$ peak. Looking at the spacing between the peaks, we see that in both cases it is close to the fit for $\cos(\theta) = 1$, indicating that there is only one current jet which departs at normal incidence from the QPCs.

For completeness, we have also measured TEF over a distance of 4 μm using the same injector as in Fig. S7e but connecting the detector to the lowest QPC electrode. The result is shown in Fig. S7f and, as expected from Equation S4, the peak spacing is approximately halved with respect to Fig. S7e. The result is summarized by comparing Fig. S7k with Fig. S7l. We note, however, that in both cases the spacing obtained from the spectra is slightly larger than predicted by the model.

To study the influence of the size quantization of the QPCs on the TEF data we have also prepared some QPCs with a horn-like shape which do not show size quantization for any V_{bg} (see Figs. S8a and S8b). The TEF spectra shown in Figs. S8c and S8d looks similar to the data shown in S7 with similar spacing between peaks (Figs. S8g and S8h), and 6 peaks visible in the best case (Fig. S8c, $V_{\mathrm{bg}}= +3$ V). Near the CNP, quantum interference features, similar to those observed in Figs. S8a and S7, are observed indicating that size quantization at the QPCs does not play an important role in the TEF spectra. As shown in Figs. S8c and S8d, inset, the electrodes used for TEF in the C3 and C4 geometris are also rotated by

30°. We thus believe that the close similarity between the TEF spectra obtained in both cases indicates that trigonal warping does not play a dominant role in our measurements.

A. Reciprocity

To confirm that the TEF measurements are in the linear response regime we have measured $R_{\rm nl}$ in C1 ($R_{\rm nl}^{\rm C1}$) and its reciprocal geometry (C1R, $R_{\rm nl}^{\rm C1R}$), obtained by swapping the current and voltage leads [18] (Figs. S9a and S9b). The measured data is shown in Figs. S9c and S9d for $V_{\rm bg}$= +3 V and −3 V, respectively. To facilitate the comparison between the signals $R_{\rm nl}^{\rm C1}$ and $R_{\rm nl}^{\rm C1R}$ we have represented $R_{\rm nl}^{\rm C1R}$ as a function of $-B$. The almost perfect overlap of the two focusing spectra in both $V_{\rm bg}$ confirms that our measurements are in the linear response regime. We attribute the small differences to electrostatic changes originated from sweeping the gate voltages.

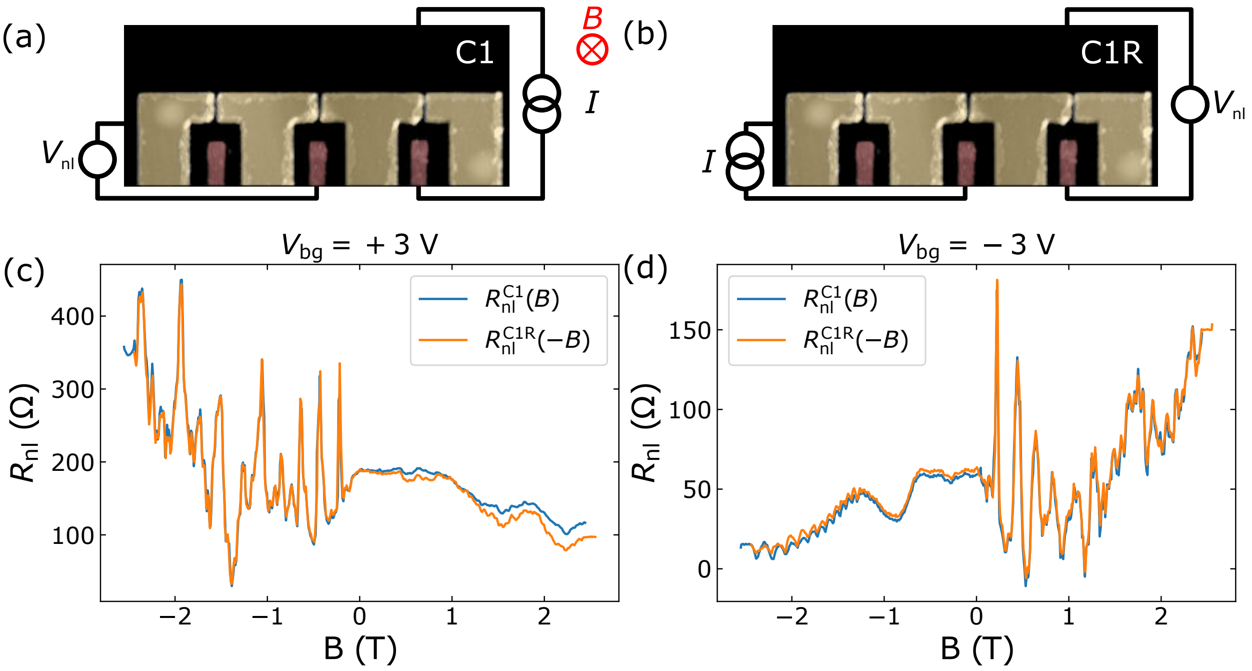

Figure S9. (a) and (b) Measurement geometries C1 and C1R used to measure the reciprocity of the TEF data, which is shown in panels (c) and (d) for $V_{\rm bg}$= +3 V and −3 V, respectively. The B has been reversed for the C1R measurements to show the agreement between both curves in a more clear way.

S7. TRANSVERSE ELECTRON FOCUSING NEAR THE CHARGE NEUTRALITY POINT

When measuring R_{nl} at $|V_{\mathrm{bg}}| < 1$ V the double-gated regions have a very small band gap and play a role in charge transport. We have measured R_{nl} vs. B in configuration C1 of the main manuscript and the results are shown in Fig. S10. In Figs. S10a and S10b, V_{tg} has been set to keep the double-gated regions at $n \approx 0$. In Fig. S10c we show R_{nl} in the presence of p-n junctions (orange and green lines) with both the single-gated and double-gated regions being n (red) and p doped (blue). The main result from Fig. S10c is the presence of plateaux which are clearly visible for $|B|$ as small as 0.5 T. This observation may be explained by considering that, when the top-gated regions conduct, the nonlocal geometry behaves like a Hall geometry when B deflects the injected carriers towards the detector. In this condition, if the system enters the quantum hall regime, one would expect plateaux. However, a significant amount of the observed plateaux occur at fields where the Shubnikov-de Haas oscillations are not yet well developed (Fig. S2), indicating that additional effects may be at play, such as quantum interference. In this case, oscillations superimposed on the linear Hall-like signal may look like plateaux on R_{nl}. The role of quantum interference is specially clear for the result in Fig. S10b, where the linear background is not observed and R_{nl} oscillates both for positive and negative B. We believe that the symmetric B-dependence, which is neither consistent with the BLG being electron or hole doped, can be explained considering that the top-gated regions are charge neutral and very close to the zero electric field condition, implying no bandgap opening and that they can conduct current (see color

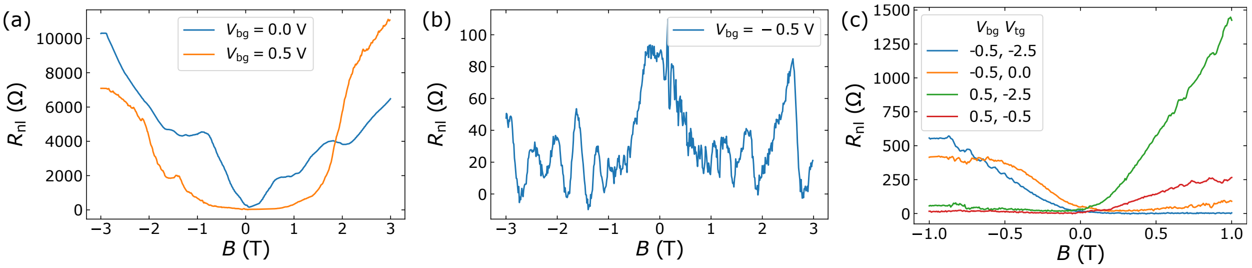

Figure S10. R_{nl} near the charge neutrality point using geometry C1. (a) and (b) were obtained setting the top-gated regions to $n = 0$ and (c) was obtained with both regions doped, inducing p-n junctions in the BLG channel (see text).

map in Fig. 1b of the main manuscript). A similar behavior is observed in Fig. S10a. In this case, an additional effect occurs: R_{nl} increases dramatically when $|B| > 1.5$ T. We believe that this is due to the formation of an insulating state in BLG near the CNP at high B [19].

S8. TRANSVERSE ELECTRON FOCUSING SAMPLE 2

We have also measured TEF in a second heterostructure. Its top and bottom hBN thicknesses are 49 and 40 nm, respectively, and its optical microscope image is shown in Fig. S11a. The QPC resistance ($R = V_{2\mathrm{p}}/I$, where $V_{2\mathrm{p}}$ is the two-terminal voltage and I the

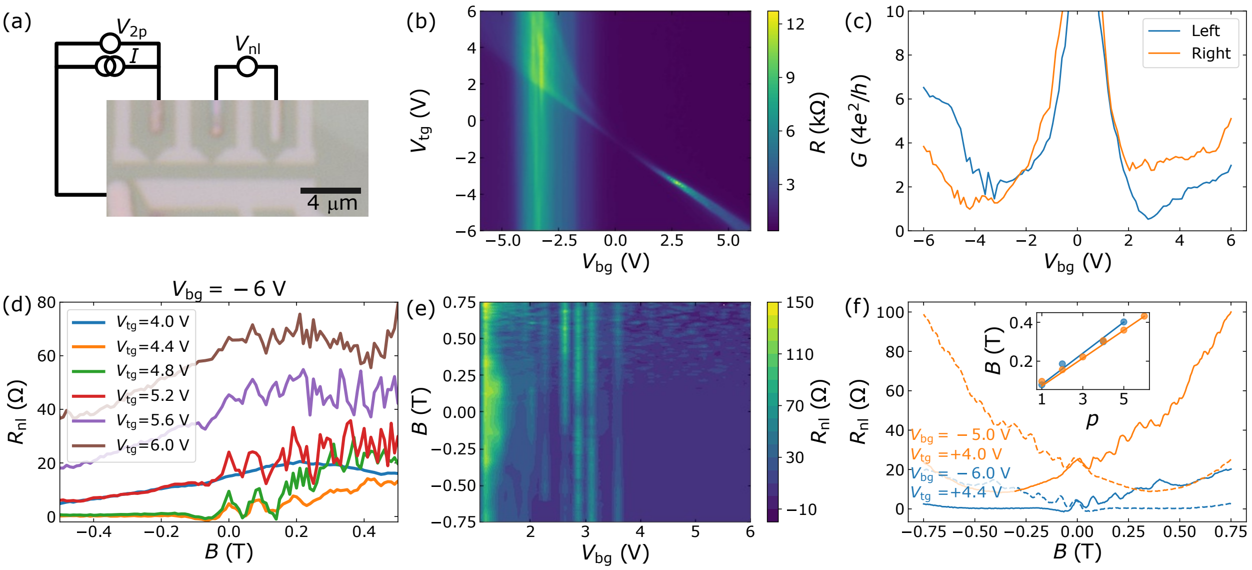

Figure S11. (a) Optical microscope image of Sample 2. The contacts to the BLG are connected to the V probes and I sources and the top gates have a horn-like shape. The measurement circuits are represented using black lines. (b) Two-point resistance $R = V_{2\mathrm{p}}/I$ as a function of V_{bg} and V_{tg}. (c) Conductance of the left and middle QPCs vs. V_{bg} extracted as in Fig. 1 of the main manuscript. (d) $R_{\mathrm{nl}} = V_{\mathrm{nl}}/I$ vs. B at $V_{\mathrm{bg}} = -6$ V and at different V_{tg}, showing clear features of TEF. (e) R_{nl} vs. B for positive V_{bg} and the V_{tg} that brings the double gated regions to the CNP. (f) R_{nl} vs. B at $V_{\mathrm{bg}} = -6$ V and -5 V. The dashed lines show the data obtained in the reciprocal geometry where we replaced the I source with the V measurement module. The inset shows the experimental (dots) and theoretical (lines) B_{f}. The latter are obtained from Equation S4 with $\theta = 0$ and n obtained from Shubnikov de Haas oscillations. The data from panels (b)-(e) was obtained at 640 mK and panel (f) at 2.4 K.

applied current, defined in Fig. S11a) is shown in Fig. S11b as a function of $V_{\rm bg}$, applied to a multilayer graphene backgate and $V_{\rm tg}$, applied to the top gates surrounding the left QPC. Following the protocol described in the main manuscript, we extracted G of the left and middle QPCs in Fig. S11a using $G = (R_{\rm max} - R_{\rm min})^{-1}$, where $R_{\rm max(min)}$ is the maximum (minimum) value of R at each $V_{\rm bg}$. The result is shown in Fig. S11c. Note that, since the hBN thicknesses are approximately two times thicker than for Sample 1, the applied $V_{\rm bg}$ and $V_{\rm tg}$ are also larger.

By monitoring $R_{\rm nl} = V_{\rm nl}/I$ vs. B, where $V_{\rm nl}$ is the nonlocal voltage, we obtained the TEF spectra shown in Fig. S11d for $V_{\rm bg}= -6$ V and at different $V_{\rm tg}$. This result shows that, even though the background is sensitive to small changes on $V_{\rm tg}$, the first TEF peak is robust against a $V_{\rm bg}= 0.2$ V change. Note that $R_{\rm nl}$ shows a peak for $B = 0$ which is not expected from TEF measurements. The results obtained for $V_{\rm bg}> 0$ are shown in Fig. S11e and, even though signatures of quantum interference can be seen for $B > 0.25$ T, no clear signs of TEF can be found.

To confirm that our measurements are in the linear response regime we performed reciprocity checks on the TEF data measured at $V_{\rm bg}= -5$ V and -6 V. The almost perfect match between both curves confirms that the bias current (100 nA) is small enough so that our measurements are in the linear response regime.

The TEF spectra shown in Figs. S11d and S11f show a peak at $B \approx 0$ which would be compatible with the $p = 1$ TEF peak if the magnet remanence $B_0 \approx -B_{\rm f}$. This hypothesis can be ruled out from the reciprocity data. A large B_0 would lead to a horizontal (B) shift of the reciprocal measurements (dashed lines) with respect to the original ones (solid lines). This is a consequence of the reciprocity theorem which states that, in a non-magnetic system and in the linear response regime, $R_{ijkl}(B) = R_{klij}(-B)$, where the first pair of indexes denote the contacts connected to the I source and the second pair the contacts used for the V measurements [18]. If B is shifted by B_0, then R_{ijkl} will coincide with R_{klij} at $B = B_0$ instead of 0. Note that both measurements were performed sweeping the magnet from 750 mT to -750 mT so the same B_0 is expected from both measurements. Thus, the coincidence of the direct and reciprocal TEF data at $B \approx 0$ confirms that $B_0 \approx 0$ and we can conclude that $R_{\rm nl}$ shows an additional peak at $B = 0$. Most likely, this peak is caused by the detection of the ballistic electron stream reflected at the opposite BLG edge.

S9. NUMERICAL SIMULATIONS

We performed semiclassical calculations of electron focusing in BLG. These calculations require previous knowledge of the angular distribution of the currents and the shape of the Fermi surface. These parameters were obtained using a tight-binding model implemented in Kwant [20]. Both calculations are explained below and the code is available at [5].

A. Tight-binding model

We implement the tight-binding model from [7, 21] which includes four hopping parameters:

$$\mathcal{H} = -\mu \sum_{n,l} c^\dagger_{n,l} c_{n,l} + \sum_{i=0}^{4} \sum_{l} \sum_{n,m\in S_i} \gamma_i c^\dagger_{m,l} c_{n,l} + \Delta \sum_{n} (c^\dagger_{n,1} c_{n,1} - c^\dagger_{n,2} c_{n,2}) \ ,$$

where $c_{n,l}$ and $c^\dagger_{n,l}$ are the annihilation and creation operators for electron states at position n and layer l, μ is the chemical potential, Δ is the layer imbalance, and the sets of hoppings S_i, with corresponding strength γ_i, are shown in Fig. S12a. Note that $\gamma_2 = 0$ in BLG [21] and is not shown in the figure.

From this model, we extract the Fermi surface used in the semiclassical calculations. To reproduce the experimental conditions, we use the displacement field and electron density corresponding to the curves with V_{bg}=+3V in Fig. 1. From the experimental data, we find the corresponding tight-binding parameters μ = 98 meV, and Δ = 84 meV. We fit the corresponding Fermi surface with the lowest Fourier component that accounts for trigonal warping. Namely,

$$k_\tau(E, \phi) = k_{F,0} + \tau \delta k \sin(3\phi + \phi_c), \tag{S5}$$

where $\tau = \pm 1$ in valley $\pm$K, ϕ is the polar angle, and $k_{F,0}$, δk, and ϕ_c are the fitting parameters. We show the computed and fitted data in Fig. S12b.

Finally, because of trigonal warping, it is incorrect to assume that the injected electrons have an uniform angular distribution. Instead, electrons are injected as two valley-polarized jetstreams from the QPC. To obtain the appropriate distribution, we compute the angular distribution of the current density from a QPC with width W_i = 7.1 nm using Kwant. The simulated device is depicted in Fig. S12c. We then fit the resulting distribution as

$$\frac{dI}{d\theta}(\theta, \theta_0, \Gamma) = \frac{I}{N} \left[G(\theta, \theta_0, \Gamma) + G(\theta, -\theta_0, \Gamma) \right], \tag{S6}$$

where $G(\theta, \theta_0, \Gamma)$ = is a gaussian distribution, θ_0 is the peak position, Γ is the width, and $N = \int_{-\pi/2}^{\pi/2} (G(\theta, \theta_0, \Gamma) + G(\theta, -\theta_0, \Gamma))d\theta$ the normalization factor. The fitted data is shown in Fig. S12d. Since each gaussian corresponds to one of the valleys, we then use the corresponding sign of $theta_0$ for each Fermi surface in Eq. S5. We also use a smaller width $(\Gamma/8)$ to obtain narrow TEF peaks as the ones observed in the measurements.

B. Semiclassical calculations

Using the Fermi surface obtained by fitting the tight-binding results with Eq. S5 (Fig. S12b, red line), we compute the electron trajectories using $\vec{r} = (x, y) = \hbar(d\vec{k}_\tau/d\phi)/(eB)$ and assuming specular reflection [9].

To obtain the TEF spectra plotted in Figs. 2e and 2f of the main manuscript we assumed that the injector is a point contact with $W_i \ll L$ and the collector has a finite width W_c. For every injection angle $-\pi/2 < \theta < +\pi/2$, we compute the electron trajectory to determine whether it will reach the detector (that is, for all $\vec{r}$ where $y = 0$, we determine if $L < x < L + W_c$). We have assumed that the current dependency on θ follows the distribution shown in Fig. S12d. Each trajectory that hits the collector adds $dI/d\theta$ to the collected current. The final result at each B is obtained by summing all the contributions in an equally-spaced distribution of $-\pi/2 < \theta < \pi/2$ multiplied by the corresponding $dI/d\theta$.

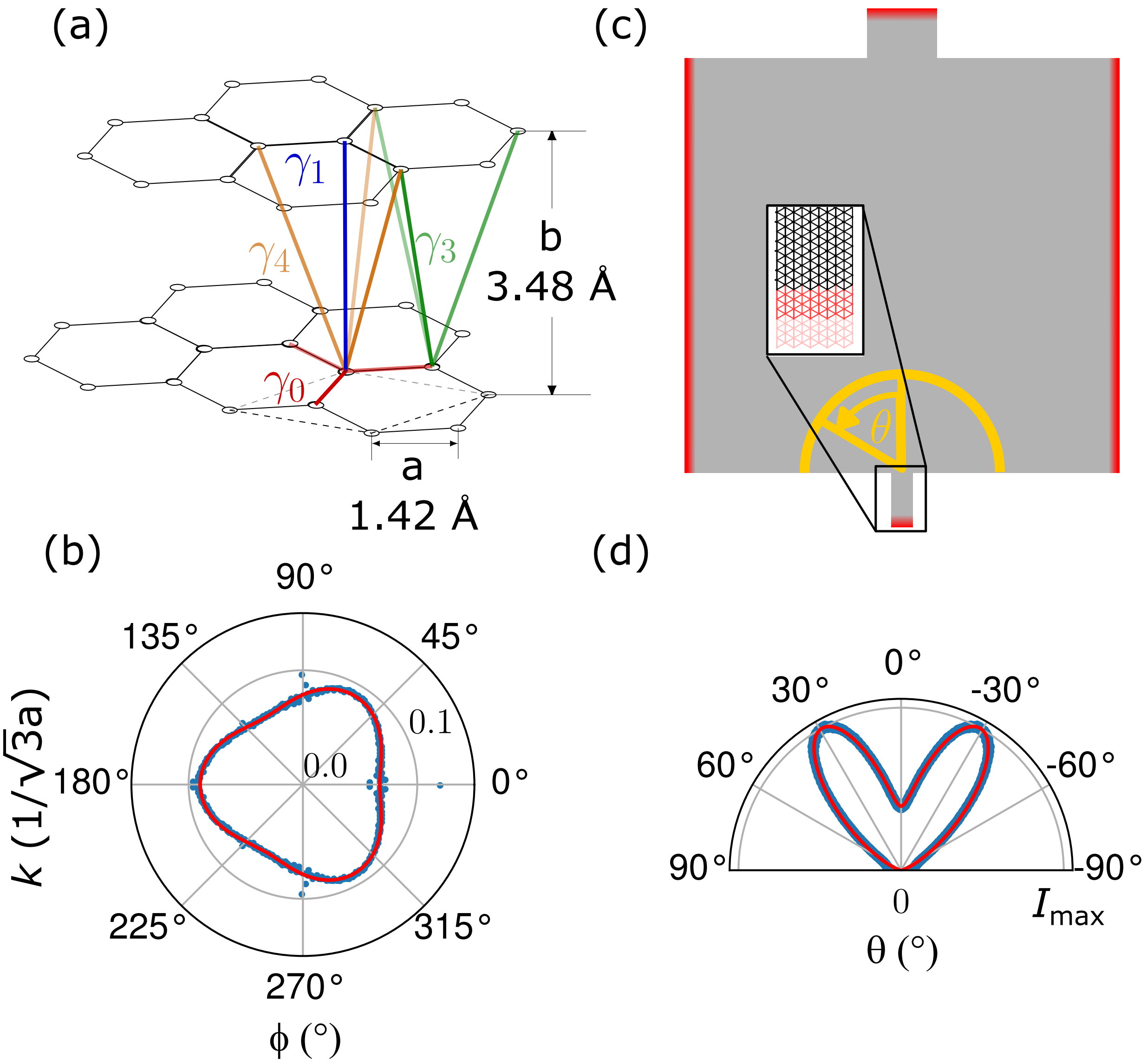

Figure S12. (a) Illustration of the BLG tight-binding model with the relevant hoping parameters γ_0 to γ_4. The unit cell is defined by the dashed lines at the lower graphene layer. (b) Computed Fermi surface (blue dots) and fit from Eq. S5 (red line). (c) Device simulated to extract the current angle distribution. Electrons are injected from the QPC in the left, and the current density is computed at the orange line. The crystallographic orientation makes $\theta = 0$ correspond to $\phi = 90°$ in panel b. The extra leads avoid backreflection of the electrons to the orange circle. The leads are shown in red. (d) Computed current distribution (blue dots) and fitted distribution from Eq. S6 (red line).

REFERENCES

[1] P. Zomer, M. Guimarães, J. Brant, N. Tombros, and B. Van Wees, Fast pick up technique for high quality heterostructures of bilayer graphene and hexagonal boron nitride, Applied Physics Letters **105**, 013101 (2014).

[2] D. Purdie, N. Pugno, T. Taniguchi, K. Watanabe, A. Ferrari, and A. Lombardo, Cleaning interfaces in layered materials heterostructures, Nature Communications **9**, 1 (2018).

[3] K. S. Novoselov, A. K. Geim, S. V. Morozov, D. Jiang, Y. Zhang, S. V. Dubonos, I. V. Grigorieva, and A. A. Firsov, Electric field effect in atomically thin carbon films, Science **306**, 666 (2004).

[4] L. Wang, I. Meric, P. Huang, Q. Gao, Y. Gao, H. Tran, T. Taniguchi, K. Watanabe, L. Campos, D. Muller, *et al.*, One-dimensional electrical contact to a two-dimensional material, Science **342**, 614 (2013).

[5] J. Ingla-Aynés, A. Manesco, T. S. Ghiasi, S. Volosheniuk, and H. S. J. van der Zant, Data underlying the publication: Specular electron focusing between gate-defined quantum point contacts in bilayer graphene, https://doi.org/10.4121/21977237 (2023).

[6] A. Laturia, M. L. Van de Put, and W. G. Vandenberghe, Dielectric properties of hexagonal boron nitride and transition metal dichalcogenides: from monolayer to bulk, npj 2D Materials and Applications **2**, 1 (2018).

[7] E. McCann and M. Koshino, The electronic properties of bilayer graphene, Reports on Progress in Physics **76**, 056503 (2013).

[8] E. Icking, L. Banszerus, F. Wörtche, F. Volmer, P. Schmidt, C. Steiner, S. Engels, J. Hesselmann, M. Goldsche, K. Watanabe, *et al.*, Transport spectroscopy of ultraclean tunable band gaps in bilayer graphene, Advanced Electronic Materials **8**, 2200510 (2022).

[9] M. Lee, J. R. Wallbank, P. Gallagher, K. Watanabe, T. Taniguchi, V. I. Fal'ko, and D. Goldhaber-Gordon, Ballistic miniband conduction in a graphene superlattice, Science **353**, 1526 (2016).

[10] T. Taychatanapat, K. Watanabe, T. Taniguchi, and P. Jarillo-Herrero, Electrically tunable transverse magnetic focusing in graphene, Nature Physics **9**, 225 (2013).

[11] E. Hwang and S. D. Sarma, Acoustic phonon scattering limited carrier mobility in two-dimensional extrinsic graphene, Physical Review B **77**, 115449 (2008).

[12] L. Molenkamp, A. Staring, C. Beenakker, R. Eppenga, C. Timmering, J. Williamson, C. Harmans, and C. Foxon, Electron-beam collimation with a quantum point contact, Physical Review B **41**, 1274 (1990).

[13] D. Bandurin, I. Torre, R. K. Kumar, M. Ben Shalom, A. Tomadin, A. Principi, G. Auton, E. Khestanova, K. Novoselov, I. Grigorieva, *et al.*, Negative local resistance caused by viscous electron backflow in graphene, Science **351**, 1055 (2016).

[14] C. Gold, A. Knothe, A. Kurzmann, A. Garcia-Ruiz, K. Watanabe, T. Taniguchi, V. Fal'ko, K. Ensslin, and T. Ihn, Coherent jetting from a gate-defined channel in bilayer graphene, Physical Review Letters **127**, 046801 (2021).

[15] A. L. Manesco and A. Pulkin, Spatial separation of spin currents in transition metal dichalcogenides, SciPost Physics Core **6**, 036 (2023).

[16] S. Neubeck, Y. You, Z. Ni, P. Blake, Z. Shen, A. Geim, and K. Novoselov, Direct determination of the crystallographic orientation of graphene edges by atomic resolution imaging, Applied Physics Letters **97**, 053110 (2010).

[17] H. Van Houten, C. Beenakker, J. Williamson, M. Broekaart, P. Van Loosdrecht, B. Van Wees, J. Mooij, C. Foxon, and J. Harris, Coherent electron focusing with quantum point contacts in a two-dimensional electron gas, Physical Review B **39**, 8556 (1989).

[18] M. Büttiker, Four-terminal phase-coherent conductance, Physical Review Letters **57**, 1761 (1986).

[19] B. E. Feldman, J. Martin, and A. Yacoby, Broken-symmetry states and divergent resistance in suspended bilayer graphene, Nature Physics **5**, 889 (2009).

[20] C. W. Groth, M. Wimmer, A. R. Akhmerov, and X. Waintal, Kwant: a software package for quantum transport, New Journal of Physics **16**, 063065 (2014).

[21] J. Jung and A. H. MacDonald, Accurate tight-binding models for the π bands of bilayer graphene, Physical Review B **89**, 035405 (2014).
